# Chemical Changes in the Broccoli Volatilome Depending on the Tissue Treatment

**DOI:** 10.3390/molecules27020500

**Published:** 2022-01-14

**Authors:** Martyna N. Wieczorek, Piotr Mariusz Pieczywek, Justyna Cybulska, Artur Zdunek, Henryk H. Jeleń

**Affiliations:** 1 Faculty of Food Science and Nutrition, Poznań University of Life Sciences, Wojska Polskiego 31, 60-624 Poznań, Poland; martyna.wieczorek@up.poznan.pl; 2 Institute of Agrophysics, Polish Academy of Sciences, Doświadczalna 4, 20-290 Lublin, Poland; p.pieczywek@ipan.lublin.pl (P.M.P.); j.cybulska@ipan.lublin.pl (J.C.); a.zdunek@ipan.lublin.pl (A.Z.)

**Keywords:** broccoli, isothiocyanates, volatiles, freezing, GCxGC-ToFMS, confocal microscopy

## Abstract

The storage of plant samples as well as sample preparation for extraction have a significant impact on the profile of metabolites, however, these factors are often overlooked during experiments on vegetables or fruit. It was hypothesized that parameters such as sample storage (freezing) and sample pre-treatment methods, including the comminution technique or applied enzyme inhibition methods, could significantly influence the extracted volatile metabolome. Significant changes were observed in the volatile profile of broccoli florets frozen in liquid nitrogen at −20 °C. Those differences were mostly related to the concentration of nitriles and aldehydes. Confocal microscopy indicated some tissue deterioration in the case of slow freezing (−20 °C), whereas the structure of tissue, frozen in liquid nitrogen, was practically intact. Myrosinase activity assay proved that the enzyme remains active after freezing. No pH deviation was noted after sample storage - this parameter did not influence the activity of enzymes. Tissue fragmentation and enzyme-inhibition techniques applied prior to the extraction influenced both the qualitative and quantitative composition of the volatile metabolome of broccoli.

## 1. Introduction

Broccoli is one of the most popular representatives of the *Brassicaceae* family. Its beneficial properties are well known; they are mainly connected to the presence of bioactive sulphoraphane isothiocyanate, which is a product of glucoraphanin enzymatic hydrolysis. There are numerous examples of recent research proving bioactive properties of isothiocyanates which suggest that *Brassica* vegetables should be included in a daily diet [1,2,3].

Like other plants, broccoli is a source of many other volatile components belonging to different chemical groups: alcohols, aldehydes, sulphides, esters, etc. [4]. Volatiles can be analysed by many available extraction techniques, among which is SPME. The SPME technique has already been used for the extraction of volatiles from plants, and was also proven to be very useful for in vivo studies on volatilome [5]. To the best of our knowledge, none of the studies on extraction and sample preparation techniques are focused on changes in such a complex matrix as plant tissue. SPME, especially in combination with comprehensive (two dimensional) gas chromatography and mass spectrometry (GC×GC-ToFMS), gained special attention in profiling volatile compounds in sensomics, volatilomics, and flavoromics studies. This was due to an unrivalled peak capacity of GC×GC systems, the spatial separation of peaks, which facilitates a better deconvolution and identification of compounds, especially when fast Time of Flight (ToF) mass spectrometers are used as detectors [6,7,8].

There are some meaningful aspects which need to be considered when one works with fresh plant tissue that are rarely discussed in technique-oriented papers. The main problem is in obtaining a “real” profile of volatile compounds from fresh plants. Especially important for a flavour study is the instability of the volatile profile which is matrix related. This is mainly associated with the activity of enzymes, which take part in the generation and release of volatiles from plant tissues. Broccoli is an example of a plant known for its bitterness and specific, sharp odour, which are often factors related to consumers’ acceptance of this vegetable as well as other *Brassica* [3,9]. In broccoli, besides the typical odour-active molecules derived from C_18_ fatty acids responsible for the typical “green” and “grassy” aroma [10,11], there are also sulfur components present as a result of myrosinase and cysteine lyase activity, mainly nitriles and isothiocyanates [12,13,14,15]. The profile of volatile compounds in broccoli (and other *Brassica*) is very dynamic, as it is formed mostly by enzymatic reactions, strongly dependent on environmental conditions (temperature, pH, presence of specific proteins), as well as physical conditions during sampling. These factors influence the overall profile of *Brassica* volatiles [13].

The goal of this paper was to evaluate changes in the volatilome of broccoli florets which may be a result of freezing as well as mechanical tissue disruption methods used for sample preparation, along with physica l and chemical methods to inhibit enzymatic changes. It was hypothesised that these factors substantially influence the profile of volatile compounds. It was monitored by means of the solid-phase microextraction (SPME) which was optimised for this type of matrix.

## 2. Results

### 2.1. Aspects Related to SPME Extraction

SPME is a predominant extraction method used for the isolation of flavour compounds and profiling volatiles. The initial step in the elaboration of the SPME extraction parameters was fiber coating selection, as this parameter significantly influences the extraction efficiency. The four most popular fibers (DVB/PDMS, CAR/PDMS, PDMS, and CAR/DVB/PDMS) were tested for isothiocyanate extraction (allyl isothiocyanate, benzyl isothiocyanate, and isobutyl isothiocyanate) and both Carboxene-containing fibers showed the highest extraction efficiency (Figure S5A—Supplementary Materials). Then, the same fibers were used to extract volatiles from blended broccoli florets to evaluate their usefulness for extraction of other important groups of broccoli volatiles. The results are shown in Figure S5B (supplementary materials). Four groups of metabolites were taken into consideration in experiments involving broccoli florets: alcohols, aldehydes, sulphides, and isothiocyanates. For esters, aldehydes, and alcohols the peak areas of detected compounds were the highest for the CAR/DVB/PDMS fiber. Only the peak area of sulphides was higher for the CAR/PDMS fiber. Based on this finding, the CAR/DVB/PDMS fiber was selected for subsequent experiments.

To elaborate the optimal temperature for SPME extraction, blended broccoli samples were extracted at 20 °C, 4 °C, 60 °C, and 80 °C for 30 min (Figure S5C, Supplementary Materials). The alcohol concentrations increased with increasing temperature from 20 °C to 60 °C. The peak area increases for aldehydes paralleled that for alcohols, and for sulphides the increment related to temperature was much less prominent than for the remaining two groups of metabolites. This suggests that sampling at 20 °C could be sufficient for the extraction of compounds belonging to these groups which would result in minimising thermal changes in the matrix. However, the only group of metabolites that required a higher extraction temperature was isothiocyanates; they were detected only at 60 °C and 80 °C. The peak area of isothiocyanates increased by 20% when the extraction temperature was changed from 60 °C to 80 °C. The increase of extraction temperature can increase the migration of isothiocyanates to the headspace, however, it can also promote the formation of isothiocyanates by thermal decomposition of glucosinolates. The increasing concentration of isothiocyanates with increasing temperature was not likely to be related to a higher myrosinase activity because higher temperatures, such as 80 °C, are too high for the enzymatic reaction. This reaction occurred before the extraction process; thus, the high temperature apparently increased the volatility of compounds occurring after the enzymatic process. The isothiocyanates considered in this experiment were isobutyl-, n-pentyl- and, hexyl isothiocyanate). The highest total peak area was achieved after extraction at 60 °C; therefore, this temperature was applied in the subsequent trials.

The next examined parameter was the extraction time. Different extraction times from 5 min to 30 min were compared. The highest peak areas of all compounds of interest were achieved after 10 min of fiber exposition, as presented in Figure S5D (supplementary materials). Moreover, the standard deviation was also at the lowest level after this short extraction time. This was probably caused by the high volatility of compounds and their rapid transfer from the matrix to the gas phase. These results showed the importance of optimising this parameter. An additional 10 min of extraction also significantly reduced the extraction time, which made it possible to reduce the total analysis time.

### 2.2. The Influence of Freezing and Thawing on the Profile of Volatile Compounds

**Figure 1:**
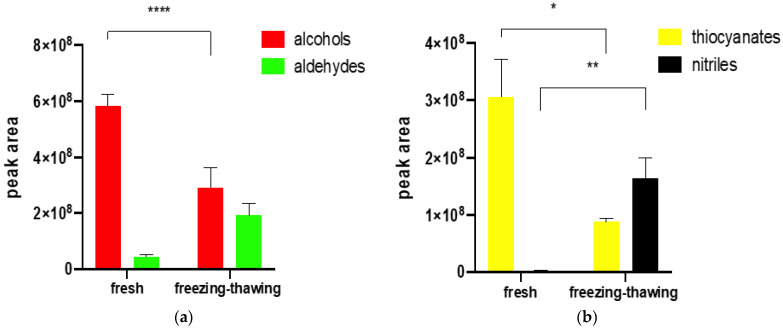
Influence of freezing-thawing of broccoli florets on the amount of extracted (**a**) LOX derived and (**b**) myrosinase derived volatiles by SPME, compared to those extracted from fresh florets. Each bar graph is expressed as mean value ± standard deviation. * *p* ≤ 0.05, ** *p* ≤ 0.01, **** *p* ≤ 0.0001 (*t*-test).

The results of the GC×GC-ToFMS analysis of fresh broccoli and the same vegetable after freezing and thawing are shown in Figure 1. The comparison of profiles of volatile compounds obtained from fresh broccoli with that of frozen and later thawed broccoli showed significant differences. In freshly cut florets, 3-hexen-1-ol, 1-hexanol dominated, and in the sample analysed after freezing and thawing, the greatest peak areas were observed for: hexanal, 2-hexanal, 2,4-heptadienal, 1-penten-3-one, 1-pentanol, as well as 3-hexen-1-ol (however, in smaller amounts than in the fresh sample) and furan 2-ethyl (Table S1). The meaningful difference related to the LOX pathway metabolites (Figure 1a). There was a change in the proportion between aldehydes and alcohols in the volatile fraction. (Figure 1a) The experiment showed that in fresh vegetables, the main products were alcohols, which were metabolites in the LOX pathway, resulting from aldehydes reduction catalysed by alcohol dehydrogenase. While in frozen/thawed samples, the main products from the LOX pathway were aldehydes: hexanal, 3-hexenal, and 2-hexenal, among others (Table S1), which suggests that alcohol dehydrogenase is effectively stopped by low temperature and there is no catalysing reduction of aldehydes after thawing. According to applied statistics, a t-test was performed and *p* = 0.00005 was observed for alcohol concentration. No meaningful probability (*p* = 0.18) was noted for aldehydes which could be caused by a high standard deviation value (Figure 1, Table S4). The profile of compounds was the same regardless of the use of liquid nitrogen or freezing at −20 °C.

The difference between samples was significant regarding the products of glucosinolates degradation, as presented in Figure 1b. In fresh vegetables, the isothiocyanates were the main products resulting from glucosinolates hydrolysis, while in frozen vegetables, the number of nitriles was growing rapidly compared to fresh tissue. The differences between isothiocyanates and nitriles in fresh and freeze-thawed vegetables were significant in both cases *p* < 0.05 (isothiocyanates: *p* = 0.02; nitriles: *p* = 0.003, according to *t*-test results) (Figure 1, Table S4)

Our results indicate that there are broader consequences of freezing-thawing on broccoli volatilome. Figure S1 (supplementary material), together with supplementary Table S1, show a more detailed insight into the profile of volatile compounds extracted from fresh and frozen-thawed broccoli florets. Some characteristic compounds for fresh and frozen-thawed broccoli tissues can be elucidated from the graph regarding peak areas (intensities) of these metabolites. For fresh broccoli, there are (Z)-3-hexene-1-ol (168), 1-hexenol (42), 3-ethyl-1, 5-octadienone (164), thiocyanic acid methyl ester (478), 1-butene, 4-isotiocyanate (36), and 3-hexenol-1-acetate (170). For frozen and thawed florets, the most abundant were 2-ethylfuran (354), 1-pentene-3-one (65), and 1-pentene-3-ol (64). The detailed list of the compounds differentiating both samples based on GC×GC-ToFMS results was listed in supplemental materials as Table S1. Compounds (489 in total) are listed alphabetically and colour-coded, where the heat scale ranges from deep blue (0 intensity) to red (highest intensities). From the comprehensive GC×GC-ToFMS data, initially, 768 features were compared for frozen-thawed and fresh broccoli. First, compounds including siloxanes, TMS derivatives, and perfluorinated compounds were discarded on the assumption that they were mainly column artifacts. Then, some of the most abundant and tailing peaks were combined, and unidentified (89) analytes were removed.

It must be remembered that the comparison was performed based on peak areas using TIC (total ion current) integration and the compounds were extracted using the SPME technique, which is based on the partition coefficients and does not provide exhaustive extraction. Though useful for comparative purposes, the comparison of peak areas does not present the absolute amount of particular compounds. For their quantitation, calibration with standards would have been required, which was not possible for that number of compounds and was also beyond the scope of this work.

### 2.3. The Confocal Microscopy Scanning Results

**Figure 2:**
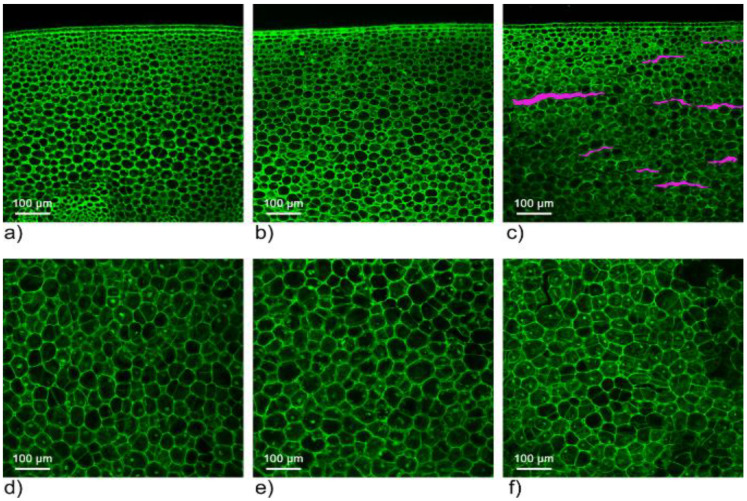
Microscopic images of broccoli tissue made in a plane perpendicular to the axis of the stem for three types of samples: (**a**,**d**) control sample, (**b**,**e**) sample subjected to rapid freezing in liquid nitrogen, (**c**,**f**) sample subjected to slow freezing (−20 °C). Characteristic tissue damage that occurs during slow freezing of samples has been highlighted in a separate colour in part (**c**).

**Figure 3:**
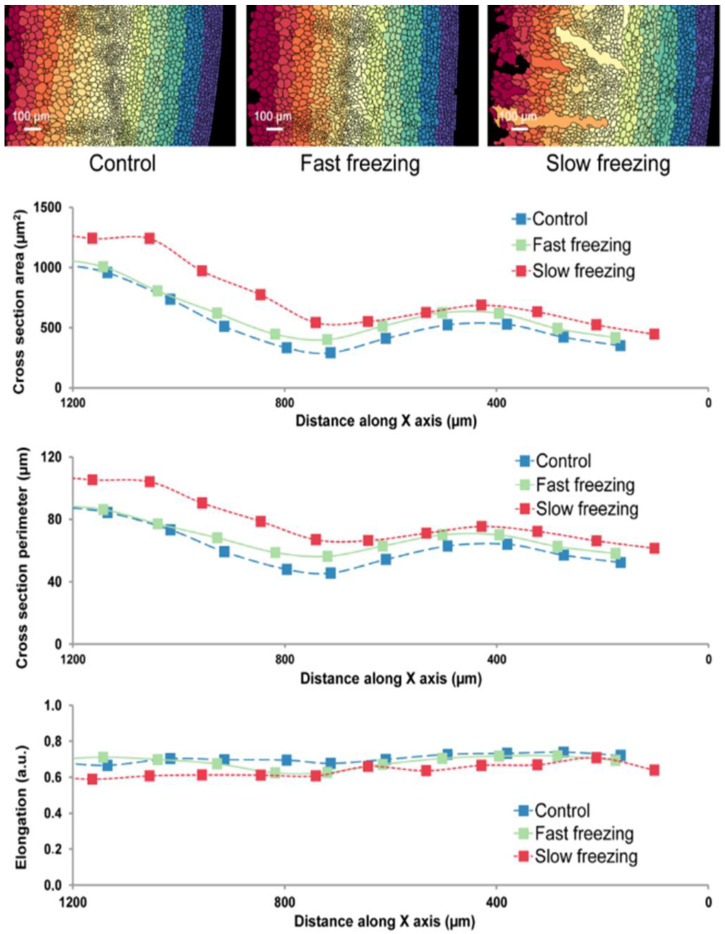
Analysis mosaic of microscopic images of tissue made of broccoli in a plane perpendicular to the axis of the stem for three types of treatment samples. For analysis, the objects were divided into groups depending on the distance from the edge of the sample (the division into groups is shown in the colour maps in the upper part of the drawing). The graphs show the average values of the geometrical parameters of the cells within the designated groups, the horizontal axis indicates the distance in micrometres from the right edge of the image (from the stem epidermis).

In order to determine the reasons for changes caused by freezing in liquid nitrogen and storage at −20 °C, first, confocal microscopy was applied, which enabled the evaluation of the state of cells in broccoli tissue. As presented in Figure 2 and Figure 3, the use of liquid nitrogen freezing did not cause significant changes in the cell structure, while in slow freezing (−20 °C) those changes were visible. As presented in Figure 3, geometrical parameters in both fast and slow freezing samples were similar to the control sample: fresh untreated broccoli (statistical data were presented in Supplementary Materials in Figures S2 and S3, Tables S2 and S3). Based on the obtained results, it is difficult to support the thesis concerning changes in profile of volatiles due to cells deterioration.

### 2.4. The pH Measurement

The pH value was measured in fresh broccoli, broccoli comminuted in liquid nitrogen, and broccoli frozen at −20 °C. The pH value did not change significantly after freezing. Differences between samples were minimal. In fresh broccoli the pH value was 6.6 ± 0.12, while in the case of the sample comminuted in liquid nitrogen it was 6,65 ± 0.17 and for the one frozen at −20 °C it was 6.56 ± 0.09. As presented, the changes in pH value after freezing did not occur, thus this parameter did not influence the enzymatic activity.

### 2.5. Enzyme Activity Assays

The changes in volatile composition after freezing in liquid nitrogen or at −20 °C could be caused by the activity of selected enzymes. In the case of broccoli, those changes were mainly related to nitrile and aldehyde concentrations. Therefore, special attention was placed on myrosinase and lipoxygenase. To check the myrosinase activity in broccoli after freezing, two trials were compared: fresh broccoli and broccoli after freezing in liquid nitrogen. The sinigrin solution was added to the enzyme extract from the vegetable because broccoli does not contain this glucosinolate; the detailed scheme is presented in Supplementary Materials, namely Figure S4. That is why broccoli could be a model matrix in which the hydrolysis of sinigrin (added to it) might be observed. The amount of glucosinolate hydrolysis products formed were measured using SPME. The zero-tests were extracts without the addition of sinigrin, in which no allyl isothiocyanate or alternative breakdown products of 3-butenonitrile sinigrin or the corresponding epitionitrile were noted.

**Figure 4:**
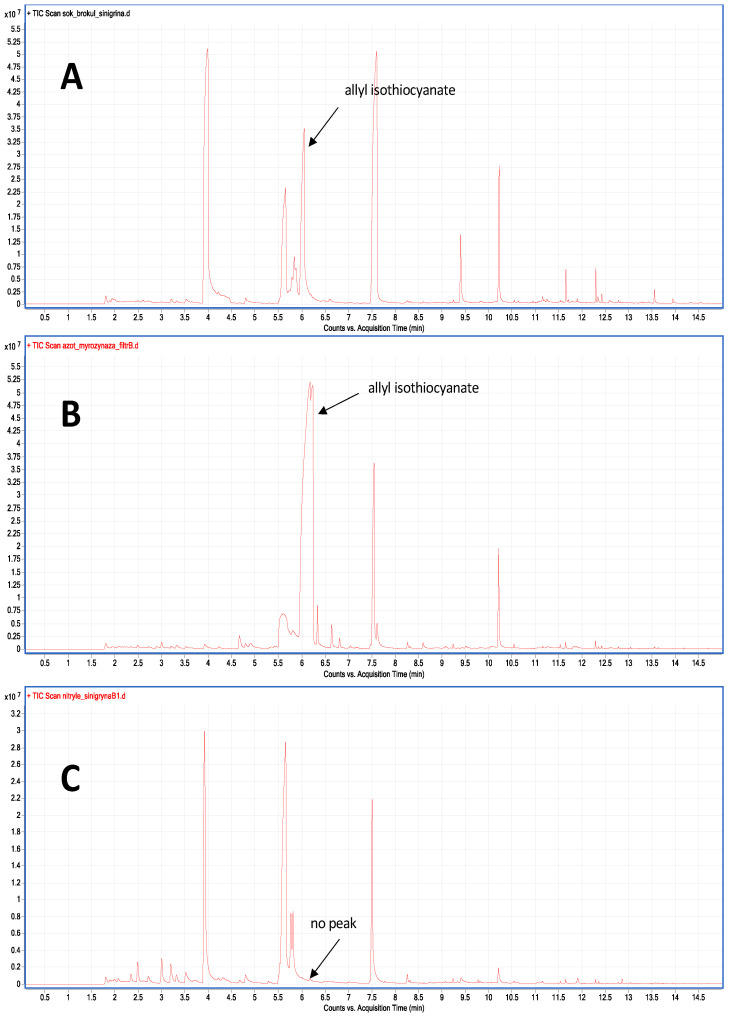
Chromatograms from enzyme activity assay experiment, presenting the amount of sinigrin degradation products-allyl isothiocyanate. (**A**) the broccoli extract before proteins separation with sinigrin addition, (**B**) extract after separation (the upper layer, with myrosinase) with sinigrin addition, (**C**) extract after separation (containing nitrile-specifier proteins) with sinigrin addition.

A significant amount of allyl isothiocyanate was found in the test with the addition of sinigrin to the extract from the fresh broccoli (Figure 4A). In the sample after freezing in liquid nitrogen, the amount of this isothiocyanate was as high as in the non-frozen sample. In the next stage, in order to separate myrosinase from epithio-specific and nitrile-specifier proteins whose mass does not exceed 50 kDa [16], AMICON filters were used to separate 100 kDa proteins, enabling separation of myrosinase (whose mass is about 200 kDa) from epithio-specific and nitrile-specifier proteins. A significant amount of allyl isothiocyanate was formed after the addition of sinigrin to the upper layer containing myrosinase (Figure 4B). The larger peak on the chromatogram was probably caused by a higher myrosinase concentration after filtration. On the other hand, after the addition of sinigrin to the lower layer, in which the nitrile-specifier proteins were present, no degradation products of glucosinolate were observed (Figure 4C).

### 2.6. The Influence of Cutting on the Profile of Volatile Compounds

**Figure 5:**
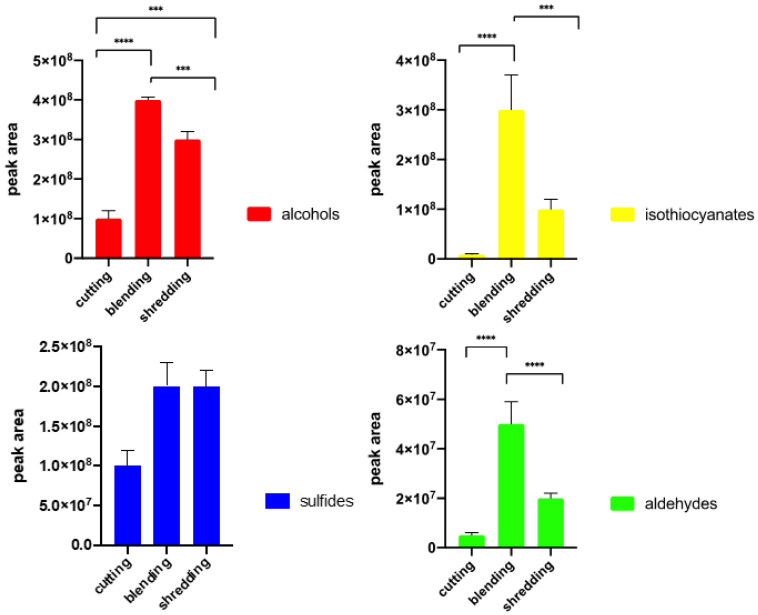
Influence of broccoli florets preparation on the number of extracted volatiles by SPME. Each bar graph is expressed as mean value ± standard deviation. *** *p* ≤ 0.001, **** *p* ≤ 0.0001 (according to the ANOVA test—Turkey’s test).

Samples of broccoli were prepared in the following three ways prior to sampling: cutting, blending, and shredding. The method of the broccoli tissues’ fragmentation had a substantial influence on the profile of the volatile compounds extracted using the SPME technique (Figure 5). After comparing the abundances of compounds in the tested main groups of volatiles, it was evident that tissue cutting influenced the overall profile of volatiles and their total amount. Though alcohols were the prevailing group in all methods, the number of aldehydes and the ratio of aldehydes to alcohols varied from 0.04 in cutting to 1.44 in blending (*p* = 0.000095, ANOVA, Tukey’s test). Shredding resulted in a ratio of aldehydes to alcohols of 0.07 (shredding-blending *p* = 0.0006; shredding-cutting *p* = 0.06, ANOVA, Tukey’s test). The number of released sulphides was the lowest for cutting and highest for shredding, however, no significant difference was noted according to the ANOVA test (*p* > 0.05). The highest differences in amounts of extracted compounds were noted for isothiocyanates. Their amount obtained after blending was 31 times higher than in the case of cutting (*p* = 0.00081, ANOVA, Tukey’s test) and almost 3 times higher than after shredding (*p* = 0.005, ANOVA, Tukey’s test). However, the number of the identified components remained almost the same in all analysed samples. Based on the ANOVA analysis and post-hoc Turkey test, the most important differences were noted in alcohol, aldehyde, and isothiocyanate concentrations, and no statistically relevant difference for sulphide concentration was noted (see Tables S5 and S6 Supplementary Materials).

### 2.7. The Influence of Enzyme Reaction Quenching on the Profile of Volatile Compounds

**Figure 6:**
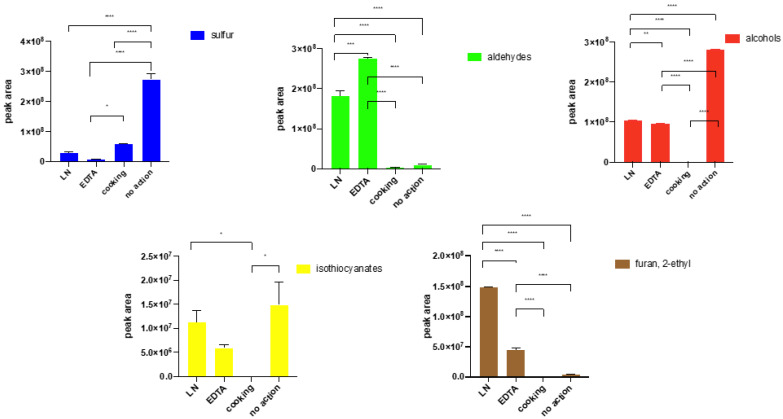
Influence of enzymatic reactions quenching in broccoli florets on extracted volatiles. Each bar graph is expressed as mean value ± standard deviation. * *p* ≤ 0.05, ** *p* ≤ 0.01, *** *p* ≤ 0.001, **** *p* ≤ 0.0001 (according to the ANOVA test—Turkey’s test).

As presented in Figure 6 and Tables S7 and S8 (supplementary materials), different inhibition techniques gave variable effects. The main groups of volatiles were selected to visualise differences between treatments. EDTA led to an increase in aldehyde concentration and, at the same time, a visible reduction in alcohol amount (comparing to the cooked sample and control sample, *p* values were below 0.0001, and in case of sample treated by liquid nitrogen < 0.001, according to Tukey’s Multiple Comparison Test—see Table S8). There was also a significant growth of 2-ethyl furan concentration in samples treated with liquid nitrogen and EDTA compared with fresh vegetables (*p* < 0.00001 in both cases, Tukey’s test). In samples treated with liquid nitrogen, sulphur and alcohol levels decreased compared to the fresh tissue (*p* < 0.0001, post-hoc Tukey’s test). However, the concentration of aldehydes was higher than in the untreated sample (*p* < 0.0001 post-hoc Tukey’s test). It is important to realise that the inhibition agent, EDTA, was introduced to the sample with water, resulting in a lowered partition coefficient of some analytes. The important observation is the low standard deviation in samples treated with EDTA which is particularly relevant in the case of working with fresh plant tissue. The liquid nitrogen treated profile of volatiles compared to fresh tissues confirm our earlier results.

## 3. Discussion

Freezing is a common practice both for the storage of vegetables for culinary purposes and for experiments in metabolite profiling or fingerprinting. Storing frozen vegetables in temperatures below 0 °C (−15 to −25 °C) is a way to preserve their nutritional value. Often, samples of vegetables for analytical purposes are also stored at this temperature range. Grinding plant tissues in liquid nitrogen is a popular and often used method prior to extracting metabolites. As shown in plant tissue, “freezing” and “defrosting/thawing” may have a significant impact on the volatilome. This indicates that in the case of living tissue, there is no possibility to compare results concerning fresh vegetables with results concerning frozen (−20 °C) and thawed vegetables due to the significant differences between the samples. The most relevant distinctions were observed in the LOX pathway metabolites and the glucosinolates and myrosinase system products. This problem is worth attention, considering the health benefits of isothiocyanates consumption.

The problem of methanethiol and dimethyl disulphide formation in crushed, homogenized, and frozen-thawed tissues of broccoli florets has been already investigated [14]. These volatiles were formed in crushed florets; however, their formation was inhibited in frozen-thawed tissues. The inhibition of DMDS production during the freeze-thawing cycle was explained as a result of a pH drop in the tissues, adherence of DMDS into the tissue surface, and a weakening of cysteine sulphoxide lyase activity under acidic conditions. With a tissue pH of 8.0 in crushed, homogenized, but most significantly in frozen-thawed tissues, DMDS dominated compared to tissues without a buffer [17].

It is known that the optimum pH for myrosinase is strongly dependent on its source. Myrosinase from Brussel sprouts was characterized by an optimum pH of 6.0–6.5, while myrosinase from white cabbage had an optimym pH of 8.0. For the enzyme from mustard or rapeseed, on the other hand, the maximum activity was noted in the pH range of 4.5–4.9 [18,19]. In the case of broccoli, which was the subject of this study, the optimal pH was between 6.5 and 7 [20]. The experiment with protein separation confirms the hypothesis that nitriles do not have an autonomic hydrolytic enzyme activity. However, this step does not explain the source of the nitriles in the broccoli sample after freezing. Moreover, it does not support the hypothesis that glucosinolates are a source of a high nitrile concentration after freezing.

In the sample preparation aspect of the study, it was shown that blending had a substantial influence on the increase of released volatile metabolites, especially isothiocyanates, aldehydes, and alcohols. As expected, the extract from more fragmented tissue gave a more abundant chromatogram. The damage of plant tissue leads to the release of a complex blend of volatiles responsible for the specific odour of disrupted plant tissue. “Green leaf volatiles,” which are mostly C6 aldehydes, alcohols, and esters, are products of the distribution of fatty acids by lipoxygenase [11]. Besides “green” aroma compounds, typical for Brassica vegetables, there are isothiocyanates and nitriles which are degradation products of glucosinolates [13].

The abundance of several groups of volatiles was monitored in fresh vegetables, vegetables with EDTA addition, vegetables frozen and ground in liquid nitrogen, and in cooked broccoli. In Figure 6, the presented chemical groups were selected based on the observed changes of peak areas after enzyme inhibition. Cooking as an enzyme-inactivation method is always effective due to thermal denaturation, the fastest way to denature proteins. However, high temperature activation leads to the thermal degradation of some components and the evaporation of many compounds during cooking. Moreover, a volatilome of cooked instead of raw broccoli was obtained, which disqualifies this method for investigation of volatiles in fresh plants. As a chelating agent, EDTA stops the activity of enzymes influenced by magnesium or calcium. Interestingly, the action of LN and EDTA, in fact, gave similar outcomes, such as a decrease of alcohols and sulfur components and, at the same time, an increase of aldehydes and 2-ethylfuran. This suggests that both factors started a similar response to the stress in the plant.

Among all volatile compounds isolated from broccoli tissues, sulphur compounds are of special interest. Firstly, due to their flavour importance and secondly, because of difficulties in sampling compared to other metabolites. In the sampling and extraction of volatiles, SPME is the dominant technique with adsorption type fibers (CAR/DVB/PDMS, CAR/PDMS, DVB/PDMS) used in the majority of research concerning flavour and volatiles [21].

As stated by Lestremau (2004) [22], the analysis of VSC (volatile sulphur compounds) is a challenging task because of the reaction between compounds, decomposition during analysis, or sorption onto the material used. In studies involving SPME, limitations were noticed due to the formation of artifacts occurring during the thermal desorption step. The possible reaction of sulphur compounds with steel acts as a catalyst, and interactions with other compounds, especially oxygenated compounds and amines, can be a source of artifacts. There are known reactions of the formation of DMDS from mercaptans during the analytes desorption from SPME fiber, where in case of increasing desorption temperature from 150 to 300 °C the DMDS formation increased from approximately 4% of the MeSH peak to 60% as a result of thermal oxidation. The decomposition of MeSH to DMDS is independent of the concentration of MeSH. When high desorption temperature is required, artifact formation seems to be unavoidable using Carboxene coating [22]. Warren et al. (2013) proposed the derivatization of thiols with *N*-phenylmaleimide, where SPME fiber was used to first derivatize by HS sampling of the solid derivatization agent [23]. However, compound-specific derivatization is an additional step in target analysis of lesser importance than in profiling studies.

## 4. Materials and Methods

### 4.1. Materials, Reagents and Standards

Broccoli florets were bought in a local grocery shop and stored in a cool room at 4 °C for no more than two days; however, each experiment was performed in one day, to reduce the possibility of changes in the volatile composition caused by the storing time. SPME fibres: CAR/PDMS, CAR/PDMS/DVB, PDMS, DVB/PDMS, 20 mL headspace vials were purchased in Supelco (Poznań, Poland). Potassium chloride, ethylene diamine tetraacetic acid (EDTA), standards of volatile compounds used for SPME evaluation (allyl isothiocyanate, benzyl isothiocyanate, and isobutyl isothiocyanate), and standards of compounds identified in broccoli florets (as indicated in the supplemental material—Table S1.) were purchased in Sigma Aldrich (Poznań, Poland). Due to the nature of the experiments carried out, the guidelines for research involving plants (especially IUCN Policy Statement on Research Involving Species at the Risk of Extinction and Convention on the Trade in Endangered Species of Wild Fauna and Flora) are not relevant.

### 4.2. The SPME Extraction Procedure

To select the best fiber-type for analysis, the fibers mentioned in the previous section were tested for their efficiency in the extraction of volatiles in headspace mode. All fibers were new and preconditioned prior to analyses according to manufacturer’s instructions. All mentioned fibers were tested first on a 1 mg/L mixture of isothiocyanates (allyl isothiocyanate, benzyl isothiocyanate, and isobutyl isothiocyanate in water), which were selected as the representation of important odour-active compounds, formed as a result of myrosinase activity in blended broccoli tissue. Additionally, the fibers were tested for isolation of volatiles from the fresh broccoli sample with a broad spectrum of volatile compounds. The broccoli was blended with water (1:1, *w*:*w*) and 3 g were put in the SPME vial. Each sample was prepared right before the extraction process, and the extraction procedure was performed manually. The whole experiment, focusing on the fiber choice in case of the fresh broccoli sample, was performed during one day.

Samples of broccoli were submitted to extraction at temperatures of 20 °C, 40 °C, 60 °C, and 80 °C. The broccoli sample was closed in a 20 mL vial and preincubated for 5 min in selected temperature, then the fiber was exposed for 30 min to this temperature. In the next experiment, the extraction time providing the highest peak areas was selected by sampling at four different time variants: 5, 10, 20, and 30 min. The effect of salting (addition of NaCl) on the extraction efficiency of volatiles was also tested by adding a salt solution to the blended broccoli. Bar graphs were prepared according to the sum of peak areas of the most abundant compounds from every analysed group. Peak areas of the following compounds were summed up: aldehydes: hexanal, 4-hexadienal, 2-hexanal, 3-hexanal, 2,4-heptadienal, benzeneacetaldehyde, 2-pentenal (E), nonanal; alcohols: 3-hexen-1-ol, 1-penten-3-ol, 1-hexanol, 1-penten-3-ol, 2-hexen-1-ol, 2-penten-1-ol, (Z)-, 3-pentanol; sulphides: dimethyl disulphide, dimethyl sulphide, dimethyl trisulphide, disulphide, methyl(methylthio)methyl, methanethiol, thiocyanic acid methyl ester; isothiocyanates: methane, isothiocyanato-, 4-methylpentyl isothiocyanate, 1-butene, 4-isothiocyanato-, 2-methylbutyl isothiocyanate, allyl isothiocyanate, isobutyl isothiocyanate, benzene, (2-isothiocyanatoethyl)-, 4-methylpentyl isothiocyanate, propane, 1-isothiocyanato-3-(methylthio), butane, 2-isothiocyanato-; nitriles: heptanonitrile, 3-butenenitrile, methallyl cyanide, pentanitrile, 4-methyl, hexanenitrile, hexenenitrile, 5-methyl, butanenitrile, 4-(methylthio)-, octanitrile, pentanenitrile and furans: furan, 2-ethyl. All experiments were performed in three replicates.

#### 4.2.1. The Effect of Tissue Disintegration Technique on Volatile Profile

In order to evaluate the influence of tissue fragmentation method on the volatile composition, three different tissue disintegration methods were tested. In the first one, broccoli was cut with a kitchen knife into approximately 0.5 cm square pieces and 3 g of the sample was analysed. In the second method, broccoli was blended to achieve a homogenous mass, then, 3 g of the pulp were analysed. The last option included shredding florets with a kitchen shredder, which allowed to get pieces (flakes approx. 1 mm × 3 mm × 8 mm) of broccoli much smaller than after knife cutting.

#### 4.2.2. The Enzyme Inactivation and Freezing-Thawing Influence on Volatile Composition

Three enzyme inhibition techniques were tested: saturated EDTA solution addition, milling in liquid nitrogen, and thermal inactivation (cooking). The results were compared with the fresh broccoli sample analysed without any enzyme inactivation step. Additionally, the effect of freezing and thawing on broccoli florets analysed fresh and stored overnight in a freezer (−20 °C) and thawed before analysis was studied. Blending was used in the case of all samples in this approach prior to the SPME extraction.

In all the above experiments, samples were run in triplicates and peak areas were compared. Each experiment was run in a single sequence with a random distribution of samples in the acquisition queue to minimize the influence of time between sample preparation and analysis. Peaks were automatically integrated with the S/N ratio set to 100:1. Results provided in Figure 2, Figure 3, Figure 4 and Figure 5 are mean peak areas with standard deviation (as the error bar) provided.

### 4.3. Instrumentation for Analyzing Volatiles

Analyses were performed using a comprehensive two-dimensional chromatography with time-of-flight mass spectrometry as a detection method (GC×GC–ToFMS). Compounds isolated by SPME were desorbed into the split/splitless injector port of the GC×GC–ToFMS system (Pegasus 4D LECO, St. Joseph, MI, USA) using an Agilent 80 CTC-type autosampler with SPME option. The GC was equipped with a DB-5 (25 m × 0.25 mm × 0.50 μm, Agilent Technologies, Santa Clara, CA, USA) as the first column and Supelcowax-10 (1.2 m × 0.1 mm × 0.1 μm, Supelco, Bellefonte, PA, USA) as the second column. The injector temperature was set at 250 °C and injection was performed in the splitless mode. For tested purge valve opening times, 0.5 min was optimal for guaranteeing high peak areas and best repeatability. The carrier gas (He) flow was set at 0.8 mL/min. The oven temperature was programmed as follows: 40 °C (1 min), 6 °C/1 min to 200 °C (0 min), 25 °C/1 min to 235 °C (5 min). Secondary oven: 65 °C (1 min), 6 °C/1 min to 225 °C (0 min), 25 °C/1 min to 260 °C (5 min). The transfer line temperature was 260 °C. The modulation time was 4 s. The ToF mass spectrometer was operating at a mass range of m/z 33–333 and the detector voltage was −1700 V at 150 spectra/s. The data were collected and processed using the LECO ChromaTOF v.4.40 software with the Statistical Compare feature. TIC peak areas were used for comparison. Spectra were compared to the NIST library (version 2.0) to tentatively identify compounds of interest. Additionally, the identity of these components was confirmed by calculating their retention indices and comparing them with literature values.

The enzyme activity assay experiment was performed using the single quadrupole GC/MS system (7890A/5975MSD, Agilent Technologies, Santa Clara, CA, USA), equipped with a DB-5 column (30 m × 0.25 mm × 0.50 μm, Agilent Technologies, Santa Clara, CA, USA). The following program was used for analysis: He flow −0.8 mL/min, oven temperature 40 °C (1 min), then 6 °C/min to 200 °C, then 25 °C/min to 280 °C (3 min). Injector temp. 250 °C, transfer line temp. 280 °C. Data were processed with Agilent MassHunter B.07.00, as above using NIST05 library.

### 4.4. Confocal Microscopy

Broccoli samples were taken from the stalk. Three separate groups of vegetables were prepared: (1) control group, (2) fast freezing- samples frozen in liquid nitrogen, and 3) slow freezing – samples kept at −20 °C for a few days. The following steps were typical for procedures used in fluorescence microscopy. First, samples were sliced in 100–200 µm thin slices, and the preparations were stained with acridine orange and rinsed in deionized water. Then the tissue was placed on a microscope slide.

In this research, a laser scanning confocal microscope FluoView300 (Olympus Corporation, Tokyo, Japan) equipped with a UPlanSApo 10×/0.40 and UPlanSApo 20×/0.75 lens was used. Recorded images were transferred to a computer with a resolution of 2048 × 2048 or 1024 × 1024. Obtained microscopic images were processed and analysed in the Matlab R2010a (MathWorks, Natick, MA, USA.) program. The statistical calculations were performed in the Statistica calculation package (StatSoft, Inc., Tulsa, OK, USA).

### 4.5. The Myrosinase and Nitrile-Specifier Protein Activity

First, a phosphate buffer with a pH of 7.00 was prepared as an isolation solution. Blended broccoli (50 g) was mixed with the above-mentioned buffer. Then, after two hours in the fridge (5 °C), the solution was passed through a Whatman filter and the filtrate was centrifuged. For placing in the SPME vial, 1 mL of the upper layer was used. The rest of the upper layer was centrifuged in AMICON centrifugal filters to separate myrosinase from nitrile-specifier proteins. Then, 1 mL filtrate from Amicons and 1 mL of the upper layer were placed in separate SPME vials. A sinigrin solution in the same amount was added to all filtrates. After 5 min of reaction, samples were placed at 40 °C and a 10-min SPME extraction was performed. Those experiments were performed for fresh broccoli and broccoli frozen in liquid nitrogen. All experiments were repeated three times. The detailed scheme of this experiment is presented in Supplementary Materials in Figure S4.

### 4.6. The pH Measurement

The pH was measured in fresh and freeze-thawed samples (in liquid nitrogen and at −20 °C). The fresh broccoli sample was blended and a pH-meter was placed inside. The sample frozen in liquid nitrogen was first flooded with liquid nitrogen, then blended. The pH was measured when the sample reached room temperature. The broccoli frozen at −20 °C was left to thaw, then was blended, and its pH was measured.

### 4.7. Statistical Analysis

In each experiment, the replicates of each group were summarized as the mean value of three replicates and standard deviation was calculated. Additionally, *t*-Student, ANOVA, and post-hoc Turkey tests were performed to evaluate the *p*-value. All calculations were performed in the R environment and Excel programs.

## 5. Conclusions

Sample preparation is the first, and one of the most important, parts of the whole analytical process and the source of the most errors. Frequently, a single sample preparation step can influence the profile of metabolites. SPME is a rapid and robust technique that enables the partition coefficient-based extraction of compounds comprising plant volatilome. Moreover, this technique enables in vitro, in vivo, and ex vivo studies which are not possible in most alternative options. However, based on our research, the SPME parameter optimisation is the first, but not the only, step in the development of methods to evaluate a plant volatilome. Equally important are all aspects related to plant tissue preparation and, specifically, tissue disintegration and temperature treatment for obtaining a representative profile of volatile compounds. As demonstrated for broccoli florets, similar differences in the profile of volatiles can be encountered in all plants in which enzymes remain active. Although for many quantitative metabolomics studies the rapid enzyme inactivation is a prerequisite to further experiments, for food volatilome profiling and flavour research an undisturbed profile of fresh volatiles is required to enable monitoring of sensory important compounds. However, our study shows a significant influence of tissue disruption on the volatilome of vegetables.

## Data Availability

Not applicable.

## Supplementary Materials

The following supporting information can be founded as following, Figure S1. Compounds extracted by SPME, characteristic for fresh and frozen-thawed broccoli florets. Figure S2. Average values of geometric parameters together with standard deviations for imaging from the parenchymatic tissue area. Figure S3. Average values of geometric parameters together with standard deviations for imaging from the epidermal tissue area. Figure S4. The scheme of enzymes activity assay experiment. Figure S5. SPME extraction parameters investigated for sampling of broccoli volatiles. Table S1. Metabolites identified in fresh and frozen-thawed florets of broccoli using SPME-GCxGC-ToFMS. Table S2. The mean values and standard deviations of the measured geometric parameters for all sample treatment methods for imaging from the parenchymatic tissue area. Table S3. The mean values and standard deviations of the measured geometric parameters for all sample treatment methods for imaging from the epidermal tissue area. Table S4. Influence freezing-thawing of broccoli florets on extracted volatiles. Table S5. Influence of broccoli florets preparation on the amount of extracted volatiles by SPME. Table S6. Statistical results for the comparison of sample pairs Table S7. Influence of enzymatic reactions quenching in broccoli florets on extracted volatiles. Table S8. Statistical results for the comparison of sample pairs.

## Sample Availability

Samples of the compounds are not available from the authors.
