# Supplementary material for: Chemical Changes in the Broccoli Volatilome Depending on the Tissue Treatment
Source: Molecules. 2022 Jan 14;27(2):500. doi: 10.3390/molecules27020500 (PMC8778298; doi:10.3390/molecules27020500)
Supplement: Supplementary file 1 [file molecules-27-00500-s001.zip › molecules-1517807-supplementary.pdf]

Chemical changes in the broccoli volatilome depending on the tissue treatment

M.N. Wieczorek, P.M. Pieczywek, J. Cybulska, A. Zdunek, H. H. Jeleń

Table S1. metabolites identified in fresh and frozen – thawed florets of broccoli using SPME-GCxGC-ToFMS

Metabolite	#	fresh broccoli peak area	RSD [%]	freezed-thawed broccoli peak area	RSD [%]
(7a-Isopropenyl-4,5-dimethyloctahydroinden-4-yl)methanol	1	76 310	29,06	134 717	24,44
(Z)-3-Heptene	2	403 152	8,10	0	0,00
1-(2,4-Dimethyl-furan-3-yl)-ethanone	3	46 390	0,00	82 075	20,14
1-(Methylthio)-2-methylbut-2-ene	4	**2 010 906**	83,04	0	0,00
1,2,4-Trithiolane	5	18 613	29,82	41 832	61,11
1,2-Benzenedicarboxylic acid, butyl 2-ethylhexyl ester	6	200 276	56,16	372 349	101,40
1,2-Ethanediol, monoformate	7	317 678	100,54	638 664	47,60
1,3,5-Cycloheptatriene	8	3 696 551	12,57	16 255 953	1,54
1,3,7-Octatriene	9	220 105	7,62	0	0,00
1,3-Benzodioxol-2-one, hexahydro-, trans-	10	**1 058 207**	7,43	**1 656 909**	23,71
1,3-Cyclobutanediol, 2,2,4,4-tetramethyl-	11	0	0,00	42 750	21,77
1,3-Cyclopentanedione, 4-methyl-	12	88 276	43,00	391 083	0,00
1,3-Dioxolane, 2-(6-octynyl)-	13	40 388	32,08	47 040	50,92
1,3-Hexadiene, 3-ethyl-2-methyl-	14	178 190	57,06	383 705	85,41
1,3-Hexadiene,c&t	15	0	0,00	158 322	47,28
1,3-Octadiene	16	93 749	31,81	55 015	33,89
1,3-Pentadiene	17	10 448 424	12,82	9 251 152	21,92
1,3-Propanediol, 2,2-dimethyl-, dinitrate	18	511 883	18,57	1 671 734	18,18
1,5-Heptadiene, 3,4-dimethyl-	19	3 875 616	59,74	232 293	35,25
1,5-Hexadien-3-ol	20	**1 061 513**	20,79	0	0,00
1,5-Hexadiene, 2,5-dipropyl-	21	66 432	42,36	21 218	5,53
1,5-Pentanediol, 3-methyl-	22	316 863	40,02	110 899	35,27
1,6-Dioxaspiro[4.4]nonane, 2-ethyl-	23	1 863 727	43,50	35 929	42,12
1,6-Dioxaspiro[4.5]decane, 2-ethyl-, (2R-trans)-	24	**84 148**	25,25	**36 273**	17,40
1,6-Octadien-3-ol, 3,7-dimethyl-, 2-aminobenzoate	25	66 037	26,82	29 892	7,83
1,6-Octadiene, 5,7-dimethyl-, (R)-	26	22 215	10,41	70 793	29,16
1,7-Nonadiene, 4,8-dimethyl-	27	752 307	79,70	321 125	0,00
1,7-Octadiene, 2,3,3-trimethyl-	28	0	0,00	160 730	23,86
11-Tricosene	29	**6 912 078**	33,10	**10 941 857**	0,00
1-Butanamine, 3-methyl-	30	337 622	17,96	431 898	86,78
1-Butanol	31	102 300	62,14	116 578	99,95
1-Butanol, 3-methyl-	32	1 612 757	24,00	0	0,00
1-Butene	33	144 121	0,00	187 961	25,57
1-Butene, 2-methyl-4-[(3-methyl-2-butenyl)oxy]-	34	0	0,00	146 088	36,98
1-Butene, 3-methyl-	35	6 265 091	8,96	2 026 782	58,79
1-Butene, 4-isothiocyanato-	36	**97 567 276**	33,85	13 035 140	44,71
1-Cyclohexene-1-acetaldehyde, 2,6,6-trimethyl-	37	37 037	78,97	42 064	19,49
1-Cyclohexene-1-carboxaldehyde, 2,6,6-trimethyl-	38	309 128	44,98	582 996	63,95
1-Decene	39	257 028	46,82	3 826 698	51,01
1-Dodecene	40	351 427	26,12	974 411	23,52
1-Ethyl-1,4-cyclohexadiene	41	1 156 324	48,10	143 703	47,25
1-Hexanol	42	**213 269 259**	13,74	**81 421 227**	0,00
1-Hexanol, 2,2-dimethyl-	43	2 510 920	8,35	1 227 060	0,00
1-Hexene, 2,5-dimethyl-	44	138 096	5,68	221 994	0,00

1-Hexene, 3,5,5-trimethyl-	45	868 458	9,46	471 925	28,05
1-Hexene, 4-methyl-	46	152 307	0,00	75 350	28,78
1-Hexene, 6-bromo-	47	213 578	38,13	245 036	32,20
1H-Indene, 1-ethylidene-	48	16 889	28,23	35 824	50,06
1H-Indole, 1-methoxy-	49	54 578	91,43	64 337	41,03
1H-Pyrrole, 2,5-dihydro-	50	124 594	17,50	0	0,00
1H-Tetrazole-1,5-diamine	51	0	0,00	341 234	62,02
1-Iodo-2-methylundecane	52	211 191	0,00	125 739	97,51
1-Methoxy-2,2,3-trimethylaziridine (sin)	53	925 516	0,00	271 461	110,34
1-Methylcycloheptanol	54	179 970	41,85	617 039	70,37
1-Nonadecanol	55	143 622	13,38	70 848	0,00
1-Nonen-3-ol	56	0	0,00	4 104 686	117,70
1-Nonene	57	1 010 861	54,05	188 296	16,92
1-Octanol, 2-butyl-	58	**3 951 795**	29,66	**3 024 826**	5,49
1-Octen-3-one	59	129 970	59,70	657 101	45,98
1-Octyn-3-ol, 4-ethyl-	60	0	0,00	210 477	92,35
1-Pentanamine	61	**20 684 801**	62,13	0	0,00
1-Pentanethiol	62	69 895	60,44	0	0,00
1-Pentanol	63	2 549 622	21,91	1 959 187	52,69
1-Penten-3-ol	64	**104 982 782**	2,16	**140 224 084**	8,28
1-Penten-3-one	65	**7 092 830**	16,12	**164 701 225**	13,68
1-Pentene, 3-ethyl-3-methyl-	66	0	0,00	140 876	39,26
1-Pentene, 4-methyl-	67	37 899	12,54	281 756	35,55
1-Pentene, 5-nitro-	68	**569 152**	2,81	**998 918**	15,03
1-Propanol	69	501 357	21,25	0	0,00
1-Propene, 1-(methylthio)-, (E)-	70	**151 394**	4,15	0	0,00
1-Propene, 1-(methylthio)-, (E)-	71	48 676	21,43	0	0,00
2-(1-Cyclohexenyl)ethylamine	72	40 727	0,00	26 762	54,56
2(3H)-Furanone, 5-ethyldihydro-	73	100 970	61,43	89 965	53,80
2(5H)-Furanone, 5-ethyl-	74	**982 518**	15,63	**7 448 787**	24,09
2,2-Dimethylpropionic acid, dodecyl ester	75	10 304	12,30	16 590	0,00
2,3-Butanedione	76	2 314 645	68,22	2 793 641	116,96
2,3-Diazabicyclo[2.2.1]-hept-2-ene	77	1 032 536	25,23	0	0,00
2,3-Dihydrofuran	78	0	0,00	**645 437**	21,01
2,3-Dihydrofuran	79	43 579	44,50	295 324	107,12
2,3-Dioxabicyclo[2.2.1]heptane	80	1 196 626	28,59	0	0,00
2,3-Epoxycarane, (E)-	81	77 908	19,41	60 261	15,30
2,3-Octanedione	82	**322 877**	31,67	**7 645 564**	2,06
2,3-Pentanedione	83	51 317	44,19	1 482 209	34,59
2,4-Decadienal, (E,E)-	84	**984 500**	85,46	**575 188**	16,98
2,4-Heptadienal, (E,E)-	85	**12 552 401**	24,70	**52 185 075**	5,80
2,4-Heptadienal, (E,E)-	86	6 332 618	86,12	37 127 788	30,88
2,4-Hexadien-1-ol	87	**424 810**	53,23	**358 694**	55,49
2,4-Hexadienal, (E,E)-	88	**3 053 102**	4,02	**37 147 258**	35,60
2,4-Nonadienal, (E,E)-	89	42 233	0,00	262 875	26,92
2,4-Pentadienenitrile	90	0	0,00	548 318	35,77
2,5-Furandicarboxaldehyde	91	0	0,00	103 142	18,03
2,6-Bis(1,1-dimethylethyl)-4-(1-oxopropyl)phenol	92	70 011	6,87	120 524	40,34
2,6-Nonadien-1-ol	93	35 163	71,79	0	0,00
2,6-Nonadienal, (E,Z)-	94	151 690	54,76	546 706	18,87
2,6-Octadiene, 4,5-dimethyl-	95	**2 107 036**	5,19	465 649	38,02

2,6-Octadiene, 4-methyl-	96	261 221	6,08	0	0,00
2-Butanone, 1-bromo-3,3-dimethyl-	97	55 433	30,72	216 243	3,63
2-Butanone, 3-methyl-	98	27 842	139,46	6 013	131,09
2-Butenal	99	1 621 103	27,74	7 629 601	36,16
2-Butene, (Z)-	100	106 054	11,20	129 251	62,79
2-Butene, 1-bromo-3-methyl-	101	**8 564 658**	11,12	**199 450**	25,15
2-Butenoic acid, 3-hexenyl ester, (E,Z)-	102	1 831 817	114,47	0	0,00
2-Butenoic acid, hexyl ester	103	415 799	91,65	0	0,00
2-Chloro-2-propen-1-ol	104	147 603	55,95	0	0,00
2-Cyclohexen-1-ol, 3,5,5-trimethyl-	105	27 125	28,15	176 816	61,54
2-Cyclohexen-1-one, 3,5,5-trimethyl-	106	**3 992 133**	19,51	**6 347 346**	10,57
2-Decanol	107	223 716	65,42	962	0,00
2-Decenal, (Z)-	108	35 106	84,25	4 209	40,13
2-Decyn-1-ol	109	31 575	0,00	36 246	14,94
2-Dodecanone	110	7 046	0,00	50 616	33,30
2-Dodecen-4-yne, (Z)-	111	99 849	33,63	54 587	98,67
2-Furanmethanol, 5-ethenyltetrahydro-ŕ,ŕ,5-trimethyl-, cis-	112	47 606	22,27	60 719	52,28
2-Heptanone	113	908 663	27,76	774 026	56,44
2-Heptanone, 6-methyl-	114	214 574	36,39	317 242	0,00
2-Heptenal, (Z)-	115	931 956	67,03	4 452 299	28,06
2-Heptene	116	1 644 201	6,56	207 044	92,16
2-Hexanone, 5-methyl-	117	190 197	24,03	67 080	50,12
2-Hexen-1-ol, (E)-	118	58 970 317	19,83	17 984 738	66,41
2-Hexen-4-yn-1-ol, (E)-	119	0	0,00	1 311 000	34,09
2-Hexenal	120	**41 149 115**	1,18	**27 643 791**	36,00
2-Hexene, 6-nitro-	121	193 469	151,48	372 848	53,40
2-Hexyl-1-octanol	122	67 264	0,00	277 432	115,17
2-Methylbutyl isothiocyanate	123	**5 817 015**	33,13	**3 261 285**	39,20
2-Methyl-n-1-tridecene	124	14 139	24,22	9 127	13,12
2-n-Butyl furan	125	268 064	22,22	268 198	39,68
2-n-Butylacrolein	126	0	0,00	547 102	104,15
2-Nitro-2-methyl-1,3-propanediol	127	40 199	16,27	12 688	4,64
2-Octanone	128	**2 437 322**	17,16	**557 400**	10,42
2-Octen-1-ol, (E)-	129	540 929	8,44	1 034 615	23,56
2-Octenal, (E)-	130	714 505	58,68	1 643 714	96,69
2-Pentanol, 2,4-dimethyl-	131	0	0,00	185 169	47,33
2-Pentanol, 3-methyl-	132	209 840	28,80	555 755	35,30
2-Pentanol, propanoate	133	39 698	11,42	489 701	0,00
2-Pentanone	134	1 255 748	18,91	470 107	0,00
2-Pentanone, 4-hydroxy-4-methyl-	135	**224 938**	6,08	**693 420**	0,00
2-Penten-1-ol, (Z)-	136	67 102 293	2,03	51 885 257	97,24
2-Penten-1-ol, 2-methyl-, (Z)-	137	94 224	119,73	0	0,00
2-Penten-1-ol, acetate, (Z)-	138	544 797	33,08	160 855	85,01
2-Penten-1-ol, acetate, (Z)-	139	52 770	12,57	0	0,00
2-Pentenal, (E)-	140	3 768 977	11,77	**15 371 549**	30,63
2-Pentenal, (E)-	141	0	0,00	252 768	33,68
2-Pentene, 3-ethyl-	142	44 043	32,31	43 795	75,32
2-Phenylpropenal	143	0	0,00	263 586	42,88
2-Propenal	144	484 597	12,37	2 719 318	15,86
2-Propyn-1-amine, N-methyl-	145	150 497	99,62	1 726 667	23,30
2-Propyn-1-ol, propionate	146	418 939	10,70	507 487	50,17

2-Undecanethiol, 2-methyl-	147	**2 533 536**	24,09	**1 462 340**	9,84
2-Undecanone, 6,10-dimethyl-	148	71 993	33,25	78 750	37,09
3,3-Dimethyl-4-(N-(2-methylbenzyl)amino)-butan-2-one	149	70 748	29,98	93 673	17,28
3,4-Decadiene	150	106 078	68,62	0	0,00
3,4-Hexanedione, 2,2,5-trimethyl-	151	1 109 239	1,20	1 344 775	29,66
3,4-Pentadienal, 2,2-dimethyl-	152	284 640	31,76	204 080	49,89
3,5-Diamino-1,2,4-triazole	153	139 754	87,91	75 606	46,69
3,5-Diisopropoxy-1,1,1,7,7,7-hexamethyl-3,5-bis(trimethylsiloxy)tetrasiloxane	154	21 818	0,00	47 250	56,75
3,5-Octadien-2-one, (E,E)-	155	511 626	22,67	3 578 598	50,30
3,5-Octadien-2-one, (E,E)-	156	367 387	9,40	2 349 679	45,67
3-Buten-2-one, 3-methyl-4-(2,6,6-trimethyl-2-cyclohexen-1-yl)-	157	13 726	45,84	22 634	34,44
3-Buten-2-one, 4-(2,2,6-trimethyl-7-oxabicyclo[4.1.0]hept-1-yl)-	158	**117 148**	14,70	**956 630**	10,02
3-Butenenitrile	159	1 993 811	32,64	34 903 057	24,71
3-Chloro-4-methoxytoluene	160	104 778	19,29	99 859	8,94
3-Cyclohexen-1-ol, 4-methyl-1-(1-methylethyl)-	161	39 483	54,89	67 773	19,50
3-Cyclohexene-1-carboxaldehyde, 1,3,4-trimethyl-	162	18 310	0,00	60 105	85,58
3-Decyn-2-ol	163	0	0,00	2 566 873	56,35
3-Ethyl-1,5-octadiene	164	**146 705 331**	1,33	**13 081 395**	19,85
3-Hepten-2-one	165	0	0,00	2 853 054	49,79
3-Heptyn-1-ol	166	7 762	5,92	68 623	64,57
3-Hexen-1-ol	167	**52 258 088**	12,13	**3 814 224**	0,00
3-Hexen-1-ol, (Z)-	168	309 194 409	39,07	73 527 236	56,50
3-Hexen-1-ol, acetate, (E)-	169	2 473 996	40,14	0	0,00
3-Hexen-1-ol, acetate, (Z)-	170	**103 141 143**	10,27	**978 093**	11,10
3-Hexen-1-ol, formate, (Z)-	171	**5 840 346**	33,35	575 008	56,04
3-Hexen-1-ol, propanoate, (Z)-	172	6 263 991	11,50	60 938	36,54
3-Hexenal, (Z)-	173	**3 251 836**	16,47	**4 781 216**	54,09
3-Hexene-2,5-diol	174	0	0,00	417 395	75,86
3-Hexenoic acid, butyl ester, (Z)-	175	**148 029**	6,55	**72 140**	24,81
3-Hexyn-1-ol	176	64 224	0,00	72 375	49,25
3-Methylpyridazine	177	14 471	0,00	621 073	58,85
3-Octyne	178	0	0,00	3 163 740	48,15
3-Pentanol	179	8 507 030	22,66	0	0,00
3-Pentanone	180	16 919 850	9,54	81 229	35,57
3-Penten-1-ol, 2,2,4-trimethyl-	181	**123 420**	11,31	**73 994**	0,00
3-Penten-1-ol, 2-methyl-	182	8 164 427	51,56	0	0,00
3-Penten-1-yne, 3-methyl-	183	840 326	18,58	97 586	83,70
3-Penten-2-one	184	0	0,00	264 037	87,09
3-Pentenal, 4-methyl-	185	0	0,00	127 005	56,25
3-Propionyloxytridecane	186	998 985	11,05	73 244	0,00
3-Vinyl-1-cyclobutene	187	272 868	17,77	90 200	22,71
4-(4-Methylpent-3-enyl)-3,6-dihydro-1,2-dithiin	188	764 167	37,61	230 843	7,61
4,4-Dimethyl-2-cyclopenten-1-one	189	26 258	53,94	1 510 226	80,38
4,6-Nonadien-8-yn-3-ol, (E,E)-	190	0	0,00	309 553	28,53
4-Bromoheptane	191	890 734	4,49	347 295	60,89
4-Decene, 2,2-dimethyl-, (E)-	192	72 401	9,47	0	0,00
4-Heptenal, (Z)-	193	47 277	49,62	309 167	20,67
4-Hexen-1-ol, (Z)-	194	141 505	60,46	68 381	16,58
4-Hydroxymandelic acid, ethyl ester, di-TMS	195	40 786	118,87	155 899	21,05

4-Methyl-1,3-pentadiene	196	8 855 639	1,20	1 875 363	0,00
4-Methyl-1,4-heptadiene	197	24 855	17,39	39 157	2,24
4-Methyl-5-decanol	198	0	0,00	5 832 752	96,69
4-Methylpentyl isothiocyanate	199	**1 843 182**	41,13	**451 011**	28,06
4-Nonene, 5-nitro-	200	**81 865**	1,43	**68 826**	19,38
4-Octanol, 4,7-dimethyl-	201	**17 071 864**	23,50	**600 762**	8,43
4-Octene, 2,2,3,7-tetramethyl-, [S-(E)]-	202	971 338	28,11	317 064	16,79
4-Penten-1-ol	203	42 634	15,15	249 497	97,79
4-tert-Butylcyclohexyl acetate	204	258 198	37,70	84 729	7,52
4-Undecene, 5-methyl-, (E)-	205	**162 253**	11,58	**149 109**	6,15
5,9-Undecadien-2-one, 6,10-dimethyl-, (Z)-	206	178 546	116,61	440 107	8,70
5-Amino-2,2-dimethylpentanol	207	0	0,00	54 131	52,94
5-Bromo-1-hexene	208	3 653 552	70,35	67 172	62,62
5-Hexen-2-one, 5-methyl-3-methylene-	209	250 160	0,00	320 373	19,88
5H-Tetrazol-5-amine	210	71 359	97,79	1 780 199	15,72
5-Tridecene, (Z)-	211	**286 427**	23,89	**129 150**	18,54
6-Methyl-2-heptyne	212	208 380	45,22	507 700	14,10
6-Octen-2-one, (Z)-	213	6 744 504	10,66	345 553	16,90
6-Oxabicyclo[3.1.0]hexane	214	242 925	58,68	333 528	71,00
7-Chlorobicyclo[4.1.0]hept-3-ene	215	156 683	60,73	0	0,00
7-Oxabicyclo[2.2.1]heptane, 1-methyl-4-(1-methylethyl)-	216	479 288	3,38	844 084	20,76
9,12-Octadecadienoic acid, methyl ester, (E,E)-	217	90 825	48,10	150 890	25,68
Acetic acid, [(aminocarbonyl)amino]oxo-	218	**29 181 765**	8,62	**29 973 993**	20,13
Acetic acid, hexyl ester	219	1 720 991	0,00	25 628	83,85
Acetic acid, methyl ester	220	651 795	20,61	262 146	4,39
Acetic acid, pentyl ester	221	250 222	24,37	32 015	47,17
Acetic anhydride	222	3 386 089	40,67	11 701 010	31,51
Acetophenone	223	512 343	31,42	1 038 219	22,44
Allyl Isothiocyanate	224	**68 282 706**	28,89	**9 772 444**	46,29
á-Pinene	225	1 630 015	3,25	1 108 520	4,87
Azeleonitrile	226	0	0,00	1 561 237	11,69
Azulene	227	138 168	24,25	349 634	25,67
Benzaldehyde	228	1 097 831	7,89	28 176 245	49,95
Benzaldehyde, 2-methyl-	229	167 619	63,47	505 101	66,33
Benzaldehyde, 4-ethyl-	230	**160 494**	27,32	**969 726**	11,27
Benzene, (1-ethyl-1-propenyl)-	231	19 431	21,59	22 016	0,00
Benzene, (1-methylethyl)-	232	**362 836**	12,09	**534 150**	2,15
Benzene, (2-isothiocyanatoethyl)-	233	2 150 897	89,81	282 646	63,52
Benzene, (2-methyl-1-propenyl)-	234	**301 405**	7,18	**396 147**	16,15
Benzene, (2-methyl-2-propenyl)-	235	31 244	70,87	74 778	23,82
Benzene, (isothiocyanatomethyl)-	236	256 950	77,64	53 555	82,65
Benzene, 1,2,3-trimethyl-	237	**601 935**	64,33	**1 019 260**	0,00
Benzene, 1,2,4,5-tetramethyl-	238	**265 991**	15,29	**185 803**	5,02
Benzene, 1,2-dimethoxy-	239	4 486 424	40,57	10 237 285	18,01
Benzene, 1,3-bis(1,1-dimethylethyl)-	240	312 659	19,22	173 517	9,93
Benzene, 1,3-diethyl-5-methyl-	241	25 505	21,65	19 950	14,45
Benzene, 1,4-diethyl-	242	37 475	59,21	86 284	4,87
Benzene, 1-ethenyl-4-ethyl-	243	118 230	10,70	156 212	12,88
Benzene, 1-ethenyl-4-methyl-	244	11 655	0,00	97 851	11,50
Benzene, 1-ethyl-2-methyl-	245	238 937	7,19	431 326	87,47
Benzene, 1-methoxy-2-methyl-	246	127 239	12,69	112 568	16,59

Benzene, 1-methoxy-4-(1-methylethyl)-	247	273 531	17,68	0	0,00
Benzene, 1-methoxy-4-methyl-	248	403 463	16,50	351 176	11,79
Benzene, 1-methyl-3-(1-methylethyl)-	249	92 385	8,95	64 568	7,69
Benzene, 1-methyl-3-propyl-	250	85 087	9,87	167 497	17,01
Benzene, 1-methyl-4-(1-methylethyl)-	251	**2 301 153**	2,38	**1 652 690**	9,71
Benzene, 2-ethyl-1,3-dimethyl-	252	180 101	0,00	136 379	1,80
Benzene, 2-propenyl-	253	13 254	33,47	32 866	14,55
Benzene, hexyl-	254	32 796	27,32	59 473	65,99
Benzene, methoxy-	255	653 300	3,91	724 205	7,65
Benzene, propyl-	256	119 218	9,34	638 254	14,34
Benzeneacetaldehyde	257	5 139 853	5,30	22 753 333	29,17
Benzeneethanamine, N-[(pentafluorophenyl)methylene]-á,4-bis[(trimethylsilyl)oxy]-	258	27 179	5,64	30 869	18,89
Benzenemethanesulfonamide	259	501 456	33,09	1 146 008	12,85
Benzenemethanol, 3,5-dimethyl-	260	31 556	11,07	21 805	21,33
Benzenemethanol, 4-(1-methylethyl)-	261	182 195	30,18	143 472	66,44
Benzenemethanol, ŕ,ŕ-dimethyl-	262	11 802	6,35	30 094	92,94
Benzenepropanenitrile	263	**139 364**	9,46	**14 795 569**	23,59
Benzenepropanoic acid, ŕ-[[(trimethylsilyl)oxy]imino]-, trimethylsilyl ester	264	51 185	30,29	49 800	43,15
Benzyl nitrile	265	354 605	32,05	865 745	5,08
Bicyclo[2.2.1]heptan-2-one, 1,7,7-trimethyl-, (1S)-	266	138 648	24,06	385 525	17,11
Bicyclo[2.2.1]heptan-3-one, 6,6-dimethyl-2-methylene-	267	11 410	33,04	19 546	10,67
Bicyclo[3.1.0]hexan-2-ol, 2-methyl-5-(1-methylethyl)-, (1ŕ,2á,5ŕ)-	268	40 450	93,15	44 501	77,75
Bicyclo[3.1.1]hept-2-ene, 2,6,6-trimethyl-, (ń)-	269	**8 775 746**	3,23	**5 400 097**	3,22
Bicyclo[3.1.1]hept-3-en-2-ol, 4,6,6-trimethyl-	270	445 837	51,78	235 541	34,52
Bicyclo[3.1.1]heptan-3-one, 2,6,6-trimethyl-, (1ŕ,2á,5ŕ)-	271	28 209	0,00	129 023	60,62
Bicyclo[4.2.0]octa-1,3,5-triene, 7-isopropyl-	272	23 036	11,86	14 608	22,67
Bicyclo[7.2.0]undec-4-ene, 4,11,11-trimethyl-8-methylene-,[1R-(1R*,4Z,9S*)]-	273	49 256	41,82	88 389	72,02
Butanal	274	276 715	1,06	1 664 318	8,35
Butanal, 2-methyl-	275	811 976	31,23	8 500 177	40,29
Butanal, 3-methyl-	276	437 320	20,05	4 234 237	34,92
Butane, 1-bromo-2-methyl-	277	**849 628**	17,61	**913 227**	45,57
Butane, 2-bromo-2-methyl-	278	581 072	45,21	0	0,00
Butane, 2-cyclopropyl-	279	0	0,00	247 413	65,44
Butane, 2-isocyanato-	280	0	0,00	156 852	63,52
Butane, 2-isothiocyanato-	281	864 003	26,94	262 334	21,20
Butane, 2-methyl-	282	2 423 928	14,25	0	0,00
Butanenitrile, 4-(methylthio)-	283	152 206	45,23	1 458 407	148,21
Butanoic acid, 2-butoxy-1-methyl-2-oxoethyl ester	284	263 481	65,39	2 550 143	51,92
Butanoic acid, 2-methyl-, hexyl ester	285	5 425 721	52,76	66 462	31,03
Butanoic acid, 2-pentenyl ester, (Z)-	286	365 437	22,50	69 390	0,00
Butanoic acid, 3-hexenyl ester, (E)-	287	**20 852 511**	1,26	**3 599 072**	20,77
Butanoic acid, hexyl ester	288	1 300 933	9,74	24 514	10,99
Butyric acid, 1-propylpentyl ester	289	59 272	0,00	64 723	32,66
Camphene	290	73 190	0,00	154 574	26,41
Carbon disulfide	291	**14 622 611**	10,41	**6 744 663**	29,13
cis-3-Hexenyl-ŕ-methylbutyrate	292	27 920	31,59	8 645	9,23
cis-Pinen-3-ol	293	55 193	8,18	0	0,00
Cyclobut-1-enylmethanol	294	204 107	49,21	388 989	26,49
Cyclododecene, (E)-	295	47 791	52,38	0	0,00

Cyclohexa-1,3-diene, 5,6-diethyl-	296	0	0,00	422 984	36,04
Cyclohexane, 1-ethenyl-1-methyl-2,4-bis(1-methylethenyl)-, [1S-(1ŕ,2á,4á)]-	297	117 668	25,45	78 367	15,71
Cyclohexane, chloro-	298	4 371 024	23,80	112 433	103,74
Cyclohexanol, 1-methyl-4-(1-methylethenyl)-, acetate	299	24 036	79,20	45 671	20,40
Cyclohexanol, 2,4-dimethyl-	300	89 502	132,27	103 227	76,04
Cyclohexanol, 5-methyl-2-(1-methylethyl)-, [1S-(1ŕ,2á,5á)]-	301	35 061	28,30	159 556	15,82
Cyclohexanone, 2,2,6-trimethyl-	302	880 780	0,00	402 278	17,54
Cyclohexanone, 2,2-dimethyl-5-(3-methyloxiranyl)-, [2ŕ(R*),3ŕ]-(.+-.)-	303	56 590	33,61	183 865	44,83
Cyclohexanone, 5-methyl-2-(1-methylethyl)-	304	51 634	19,30	142 632	0,00
Cyclohexene, 1-methyl-4-(1-methylethylidene)-	305	**1 241 117**	4,40	**678 302**	25,42
Cyclopentane, (methylthio)-	306	515 464	75,36	23 076	0,00
Cyclopentane, 1,3-dimethyl-2-(1-methylethenyl)-, (1ŕ,2ŕ,3á)-	307	60 809	5,50	125 615	9,46
Cyclopentane, 1-hexyl-3-methyl-	308	0	0,00	65 465	51,74
Cyclopentane, 1-methyl-3-(2-methylpropyl)-	309	15 812	11,76	69 202	100,00
Cyclopentane, 2-ethylidene-1,1-dimethyl-	310	134 554	16,38	70 106	72,21
Cyclopentane, bromo-	311	**4 143 192**	8,80	**22 598**	0,00
Cyclopentane, methyl-	312	0	0,00	1 843 910	26,29
Cyclopentane, nitro-	313	228 776	52,53	0	0,00
Cyclopentyl isothiocyanate	314	71 584	44,48	244 717	21,21
Cyclopropane, 1-ethyl-2-methyl-, cis-	315	84 067	81,01	0	0,00
Cyclopropane, 2-(1,1-dimethyl-2-propenyl)-1,1-dimethyl-	316	125 248	46,68	0	0,00
Cyclopropane, ethyl-	317	**2 280 261**	1,45	**1 325 472**	0,00
Cyclopropane, isothiocyanato-	318	**2 627 821**	43,82	**198 424**	0,00
Cyclopropane, propyl-	319	**2 472 696**	6,94	**186 817**	0,00
Cyclopropane, tetramethylpropylidene-	320	20 665	112,97	2 910	25,16
Cyclopropane-1,1,2,2-tetracarbonitrile, 3-ethyl-3-methyl-	321	976 739	30,97	1 331 919	32,15
Decanal	322	474 683	50,96	972 082	25,87
Decane	323	**633 497**	47,17	**1 050 716**	32,70
Decane, 2,6,7-trimethyl-	324	469 895	7,51	634 454	72,74
Diallyl carbonate	325	12 931 002	10,40	36 143 905	49,37
Diazene, dimethyl-	326	176 959	7,77	0	0,00
Dibenzo[cd,g]indazole-3-sulfonic acid, 2,6-dihydro-6-oxo-	327	835 265	155,33	3 056 982	134,42
Dibutyl phthalate	328	300 679	64,62	1 014 917	74,47
Dihydromyrcene	329	100 798	25,23	431 121	63,88
Dimethyl sulfide	330	7 817 123	9,91	5 601 075	7,20
Dimethyl trisulfide	331	**67 002 388**	18,34	**19 480 654**	59,11
Disulfide, dimethyl	332	**29 361 807**	6,95	**18 615 463**	36,71
Disulfide, methyl (methylthio)methyl	333	20 351 960	39,11	277 454	45,34
Disulfide, methyl 2-propenyl	334	224 124	28,36	57 523	41,53
Disulfide, methyl propyl	335	480 120	52,66	0	0,00
Dodecanal	336	**202 545**	106,01	**200 816**	43,40
Estragole	337	111 826	37,09	116 223	6,41
Ethane, (methylthio)-	338	398 025	60,98	0	0,00
Ethanol, 2-(ethylthio)-	339	180 216	39,80	7 861	0,00
Ethanol, 2-nitro-	340	929 247	53,68	0	0,00
Ethanone, 1-(3-ethyloxiranyl)-	341	0	0,00	19 142	48,71
Ethanone, 1-(3-methylphenyl)-	342	223 647	29,86	340 830	13,36
Ethenamine, N-methylene-	343	398 361	10,76	595 921	4,64
Ether, tert-butyl isopropylidenecyclopropyl	344	4 070 573	5,34	92 386	0,00
Ethyl Acetate	345	11 134 434	59,22	814 862	9,45

Ethylbenzene	346	292 225	12,40	2 237 726	10,30
Eucalyptol	347	795 274	9,11	1 467 422	11,37
Formic acid, ethenyl ester	348	45 206	9,32	77 545	0,00
Furan	349	163 457	22,46	443 151	58,10
Furan, 2,3-dihydro-4-(1-methylpropyl)-, (S)-	350	63 499	43,70	42 676	68,44
Furan, 2,3-dihydro-5-methyl-	351	0	0,00	145 151	27,59
Furan, 2-butyltetrahydro-	352	79 338	27,81	430 348	110,74
Furan, 2-butyltetrahydro-	353	0	0,00	1 085 706	41,74
Furan, 2-ethyl-	354	17 833 776	14,38	183 486 875	24,18
Furan, 2-ethyl-5-methyl-	355	60 408	0,00	57 306	54,12
Furan, 2-methyl-	356	**2 973 139**	15,91	**1 451 687**	28,59
Furan, 2-pentyl-	357	13 476 195	36,39	11 324 854	33,05
Furan, 2-propyl-	358	365 505	7,60	47 506	72,20
Furan, tetrahydro-	359	6 577 187	15,11	7 934 173	18,81
Furan, tetrahydro-2-(methoxymethyl)-	360	59 563	12,96	0	0,00
Furan, tetrahydro-2-methyl-	361	0	0,00	1 809 060	21,74
Heptanal	362	879 317	55,36	6 069 692	30,06
Heptane	363	327 730	16,71	3 880 199	149,39
Heptane, 2,4-dimethyl-	364	2 475 820	61,37	672 082	36,65
Heptane, 3-ethyl-	365	36 491	29,99	53 756	43,73
Heptanonitrile	366	0	0,00	27 960 300	6,05
Hex-3-ene-1,6-diol	367	162 619	0,00	984 072	18,04
Hexadecanal	368	**48 528**	50,68	**462 003**	37,99
Hexadecane	369	**252 142**	29,72	**129 557**	62,95
Hexanal	370	**23 494 988**	13,87	**58 243 384**	102,72
Hexanal, 4-methyl-	371	48 438	24,55	1 446 898	75,98
Hexane	372	2 826 917	18,11	7 106 369	53,05
Hexane, 1-(methylthio)-	373	317 379	109,49	0	0,00
Hexane, 1-isocyanato-	374	51 118	6,33	197 435	12,76
Hexane, 1-isothiocyanato-	375	795 607	80,73	46 664	55,96
Hexane, 4-ethyl-2-methyl-	376	**1 940 020**	39,20	**3 443 088**	35,73
Hexanenitrile	377	0	0,00	**28 664 520**	10,61
Hexanenitrile	378	0	0,00	6 177 534	13,88
Hexanenitrile, 5-methyl-	379	0	0,00	15 692 741	5,11
Hexanoic acid, 2-hexenyl ester, (E)-	380	364 229	37,25	68 891	18,82
Hexanoic acid, 3-hexenyl ester, (Z)-	381	**1 226 542**	36,78	**149 834**	13,64
Hexanoic acid, hexyl ester	382	73 125	57,05	0	0,00
Hexanoic acid, methyl ester	383	94 335	30,94	394 005	76,18
Indane	384	24 490	0,00	80 328	11,46
Isobutyl ether	385	118 768	16,44	49 271	43,75
Isobutyl isothiocyanate	386	4 260 696	49,08	616 970	62,67
Isopropyl isothiocyanate	387	118 070	32,83	42 098	23,90
L-Fenchone	388	369 709	29,36	349 472	12,22
Lilac alcohol D	389	**110 435**	15,45	0	0,00
Limonene	390	3 225 995	16,52	2 247 280	71,89
L-Valine, N-glycyl-	391	145 345	15,49	37 481	27,01
Mercaptoacetone	392	833 605	31,19	0	0,00
Methacrolein	393	341 025	47,73	**440 781**	6,41
Methacrolein	394	0	0,00	100 479	98,76
Methallyl cyanide	395	223 886	20,44	22 925 968	18,79
Methane, (methylsulfinyl)(methylthio)-	396	389 778	45,96	0	0,00

Methyl ethyl disulfide	397	576 494	61,54	52 593	65,04
Methyl formate	398	**1 514 699**	35,94	**4 246 023**	29,16
Methyl pentyl disulfide	399	811 530	91,39	52 042	59,81
Monoethyl carbonotrithioate	400	612 819	65,44	332 226	47,45
Monomethyl carbonotrithioate	401	166 169	10,24	98 809	85,50
N,N'-Bis(2,6-dimethyl-6-nitrosohept-2-en-4-one)	402	0	0,00	188 253	32,54
Naphthalene, 1,2,3,5,6,7,8,8a-octahydro-1,8a-dimethyl-7-(1-methylethenyl)-, [1R-(1ŕ,7á,8ať)]-	403	84 537	26,30	93 146	11,11
n-Caproic acid vinyl ester	404	0	0,00	60 014	66,29
Nonanal	405	3 116 710	42,32	5 199 982	19,73
Nonanal, 3-(methylthio)-	406	1 252 025	129,29	123 616	27,44
Nonane	407	1 037 579	61,14	417 114	42,55
Nonane, 2-methyl-	408	66 474	18,26	130 219	83,98
Nonane, 3,7-dimethyl-	409	105 357	67,42	54 901	41,24
Nonane, 4-ethyl-5-methyl-	410	51 524	0,00	106	22,67
n-Pentyl isothiocyanate	411	1 390 511	69,86	90 519	53,81
n-Valeric acid cis-3-hexenyl ester	412	42 582 022	41,53	7 165 007	34,53
Octanal	413	288 706	30,63	1 415 234	45,11
Octane, 2,6-dimethyl-	414	18 855	8,15	68 436	59,92
Octane, 4-chloro-	415	97 464	85,69	187 890	53,69
Octanenitrile	416	66 551	0,00	**238 330**	3,83
Octanenitrile	417	0	0,00	64 578	13,58
Octanoic acid, ethyl ester	418	131 096	96,92	0	0,00
Oxalic acid	419	632 485	38,17	0	0,00
Oxalic acid, allyl decyl ester	420	29 145	96,44	210	173,21
Oxalic acid, allyl dodecyl ester	421	406 224	34,23	**297 092**	29,80
Oxalic acid, allyl dodecyl ester	422	58 946	38,00	9 540	106,06
Oxalic acid, allyl hexadecyl ester	423	245 986	44,49	163 621	11,92
Oxalic acid, allyl octadecyl ester	424	375 166	72,97	127 431	127,70
Oxalic acid, dodecyl hexyl ester	425	10 620	6,80	7 459	10,24
Oxalic acid, isobutyl hexadecyl ester	426	193 613	54,59	252 622	0,00
Oxalic acid, isobutyl nonyl ester	427	626 355	18,33	449 490	56,07
Oxalic acid, isobutyl pentyl ester	428	477 204	2,81	349 091	64,33
Oxirane, pentyl-	429	453 075	10,76	1 640 273	17,58
Oxiranemethanol, 3-methyl-3-(4-methyl-3-pentenyl)-	430	64 272	22,94	89 107	61,81
o-Xylene	431	1 032 527	5,92	9 449 665	14,43
Pentadecane	432	3 990 881	140,12	175 176	0,00
Pentanal	433	365 651	0,00	581 931	9,25
Pentanal, 2-methyl-	434	23 107	32,12	60 900	28,54
Pentane	435	4 448 450	6,28	4 038 693	60,29
Pentane, 1-(methylthio)-	436	3 565 988	115,26	49 467	53,63
Pentane, 2,2,4,4-tetramethyl-	437	3 433 975	4,10	3 293 482	118,18
Pentane, 2-chloro-4-methyl-	438	77 432	109,40	135 316	101,21
Pentanenitrile	439	0	0,00	230 528	36,48
Pentanenitrile, 4,4-dimethyl-5-oxo-	440	222 975	89,03	104 627	55,70
Pentanenitrile, 4-methyl-	441	61 896	0,00	31 696 690	46,66
Pentanenitrile, 5-(methylthio)-	442	727 210	36,07	764 680	136,83
Pentanethioic acid, S-ethyl ester	443	105 670	37,24	94 719	25,96
Pentanoic acid, 10-undecenyl ester	444	218 095	3,50	86 510	11,46
Pentanoic acid, 2-methyl-, methyl ester	445	0	0,00	170 613	61,10
Pentanoic acid, 4-oxo-	446	39 718	24,97	222 799	23,95
Pentasulfide, dimethyl	447	363 435	24,47	133 711	96,77

Phenol, 2-ethyl-	448	537 860	50,43	599 495	30,28
Phenol, 4-(1-methylpropyl)-	449	222 232	0,00	187 311	16,48
Phenol, 4-(2-aminopropyl)-	450	162 245	37,08	0	0,00
Phenol, 4-ethyl-3-methyl-	451	17 912	38,58	26 551	72,57
Propanal	452	2 145 186	10,84	16 074 776	38,31
Propanal, 2-methyl-	453	208 883	24,54	1 623 141	41,99
Propanal, 3-(methylthio)-	454	269 347	17,56	699 959	18,25
Propane, 1-(methylthio)-	455	811 971	24,44	2 880 877	78,39
Propane, 1-(methylthio)-	456	154 043	49,52	0	0,00
Propane, 1-isothiocyanato-3-(methylthio)-	457	3 496 962	97,42	115 346	27,29
Propane, 2-bromo-	458	**2 744 352**	8,11	**8 856 996**	27,29
Propane, 2-methoxy-2-methyl-	459	173 974	122,49	685 256	88,95
Propanoic acid, 2,2-dimethyl-, anhydride with diethylborinic acid	460	5 758	0,00	65 531	51,55
Propanoic acid, 2-methyl-, 3-hydroxy-2,4,4-trimethylpentyl ester	461	773 806	35,27	7 771 954	94,27
Propanoic acid, 2-methyl-, hexyl ester	462	1 490 257	34,16	16 025	30,79
Propanoic acid, 2-penten-1-yl ester (Z)-	463	**2 644 236**	27,76	**75 594**	74,19
Propanoic acid, hexyl ester	464	2 202 718	28,40	23 627	81,83
p-Xylene	465	807 982	6,08	3 529 647	13,98
Pyrazine, 2-methoxy-3-(1-methylethyl)-	466	28 100	20,90	132 299	8,24
Pyrazine, 2-methoxy-3-(1-methylpropyl)-	467	17 687	15,34	74 402	3,30
ŕ-Phellandrene	468	76 746	16,52	40 249	39,06
Styrene	469	311 758	12,45	14 040 682	13,45
Sulfide, methyl 1-methyl-2-butenyl	470	65 578	55,34	0	0,00
Sulfurous acid, cyclohexylmethyl hexadecyl ester	471	40 563	32,52	42 143	43,78
Sulfurous acid, cyclohexylmethyl hexyl ester	472	368 810	14,43	319 190	65,52
Sulfurous acid, hexyl pentyl ester	473	161 393	41,88	40 974	13,54
Sulfurous acid, hexyl tridecyl ester	474	1 911 280	112,46	57 058	156,28
Tetradecane	475	**3 257 598**	4,84	**5 553 173**	4,44
Tetrahydrofuran, 2-hexyl-	476	232 451	0,00	58 614	11,26
Thiocyanic acid, 2-propenyl ester	477	2 057 410	135,17	21 470	27,10
Thiocyanic acid, methyl ester	478	**140 495 321**	16,18	**44 588 072**	46,48
Thiophene, 2-ethyl-	479	**25 397 292**	4,30	**49 761 734**	8,55
Thiophene, 2-methyl-	480	210 064	25,95	147 577	50,66
Thiophene, 2-pentyl-	481	356 183	42,24	198 731	34,89
Thiophene, 2-propyl-	482	33 413	27,30	0	0,00
Thiophene, tetrahydro-	483	450 739	99,67	22 456	138,26
trans-2-(2-Pentenyl)furan	484	869 195	7,90	1 214 303	12,94
trans-3-Nonen-2-one	485	0	0,00	67 274	42,50
trans-4-Decene	486	24 983	10,73	25 753	17,93
Undecane	487	40 427	19,22	25 780	0,00
Undecane, 3,5-dimethyl-	488	0	0,00	166 935	5,29
Urea	489	**1 688 414**	17,26	**9 815 486**	17,61
**Total [peak area]**		**2184741395**		**1860829650**	

.

Compounds were color-coded according to their peak areas from blue (smallest) to red (highest). Compounds with peak abundances of >150 000 000 extracted from fresh tissues were highlighted in blue, these for freezed/thawed – in red colour. Relative standard deviations (RDS) were provided in separate columns for fresh and freezed/thaved samples (n=3). Relative standard deviation (RSD) is expressed in % (SD*100/mean)

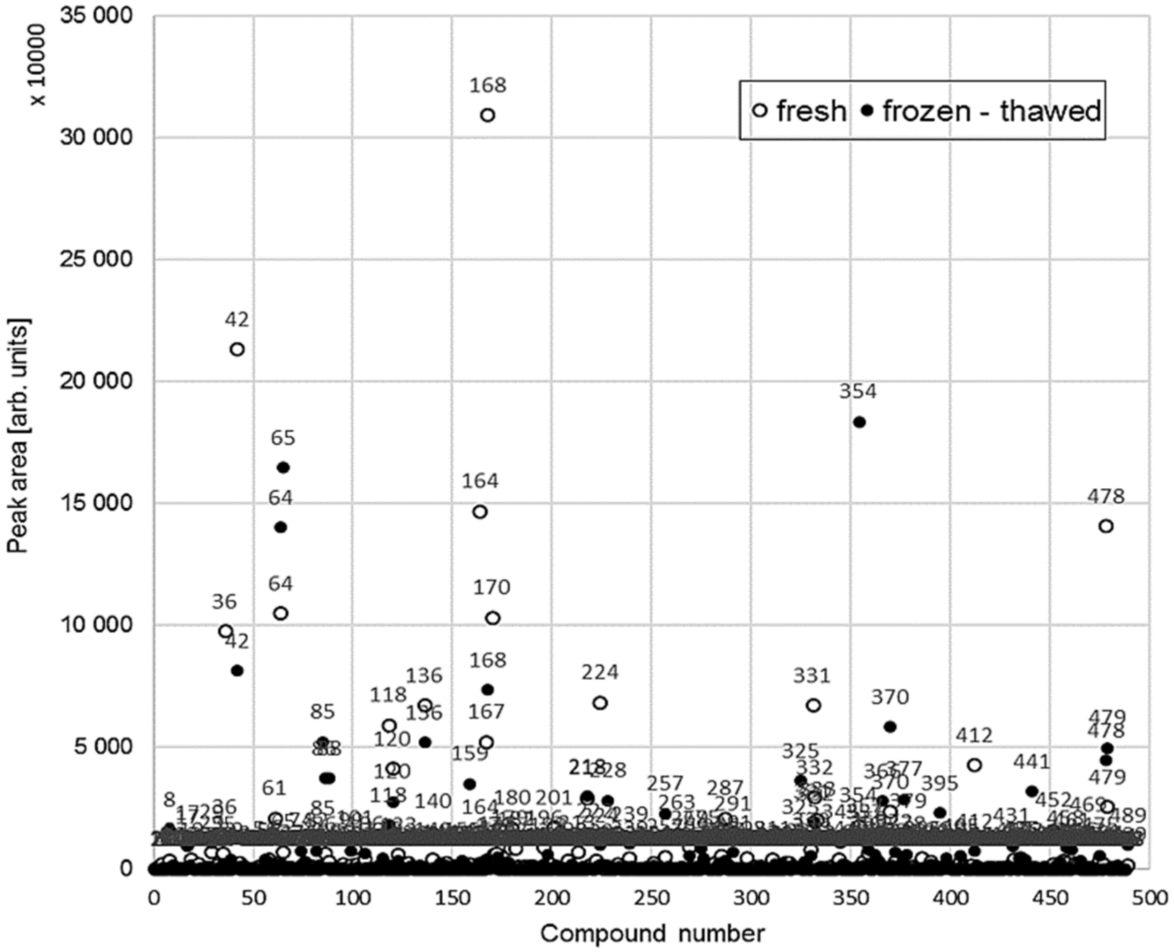

Figure S1. Compounds extracted by SPME, characteristic for fresh and frozen – thawed broccoli florets. Metabolite numbers correspond to those in Table S1 (Supplementary Material).

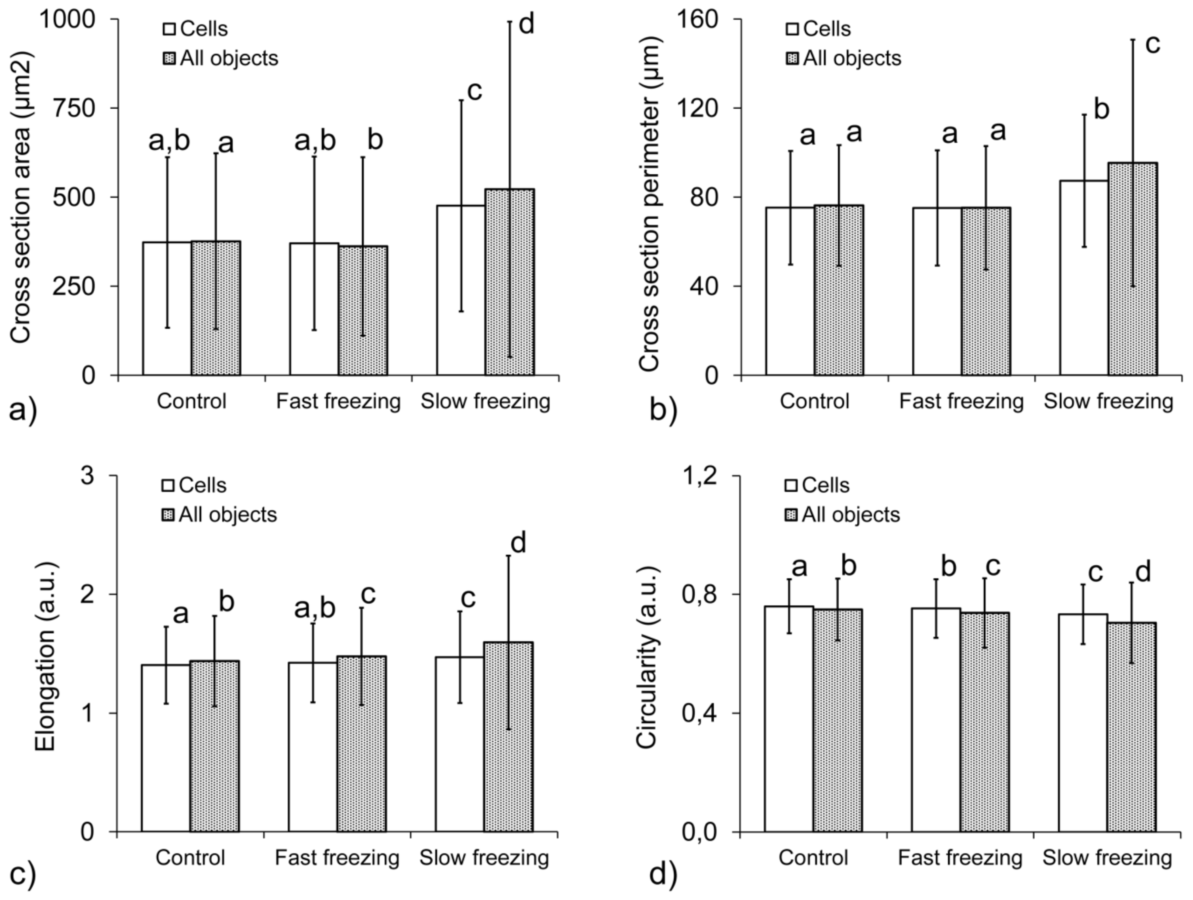

Figure S2. Average values of geometric parameters together with standard deviations for imaging from the parenchymatic tissue area. Letter designations symbolize belonging to homogeneous groups (according to the ANOVA test - Tukey's HSD test).

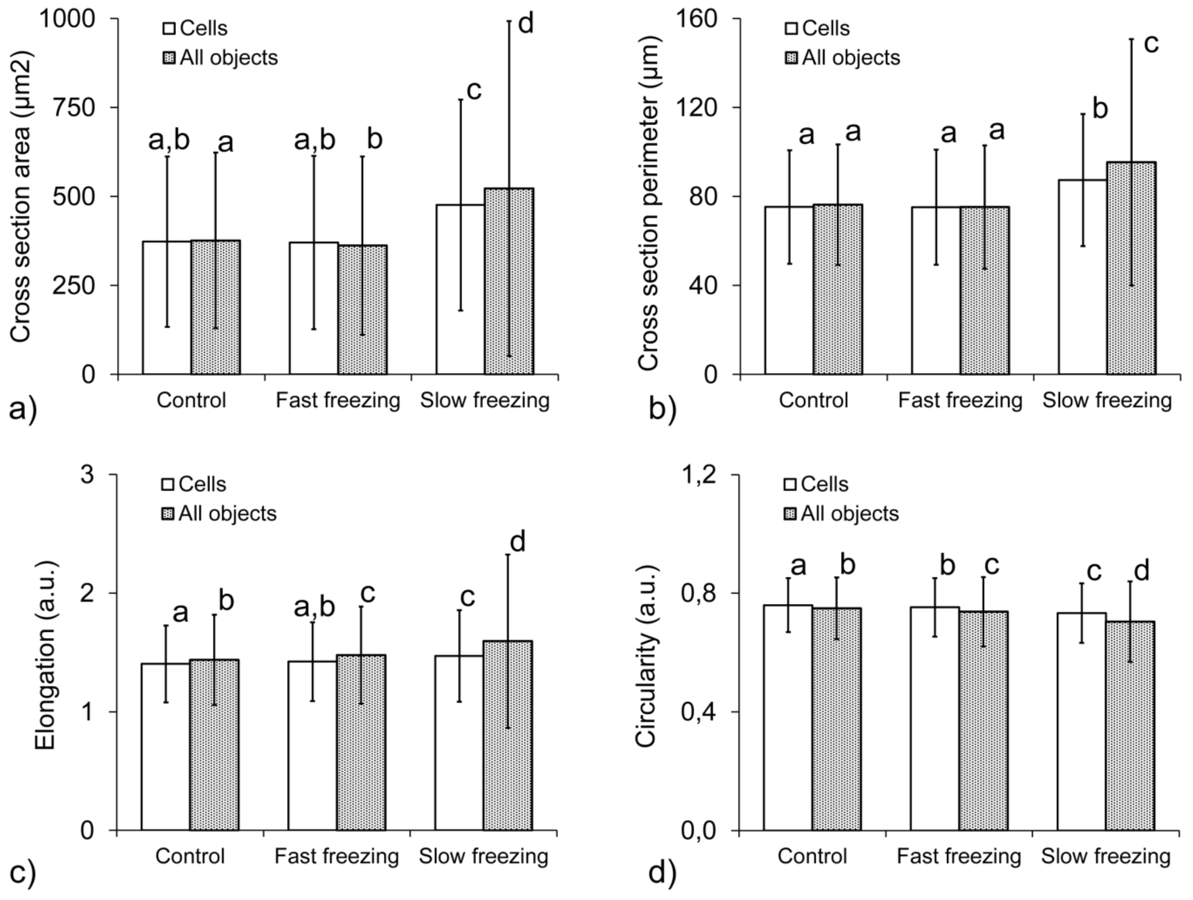

Figure S3. Average values of geometric parameters together with standard deviations for imaging from the epidermal tissue area. Letter designations symbolize belonging to homogeneous groups (according to the ANOVA test - Tukey's HSD test).

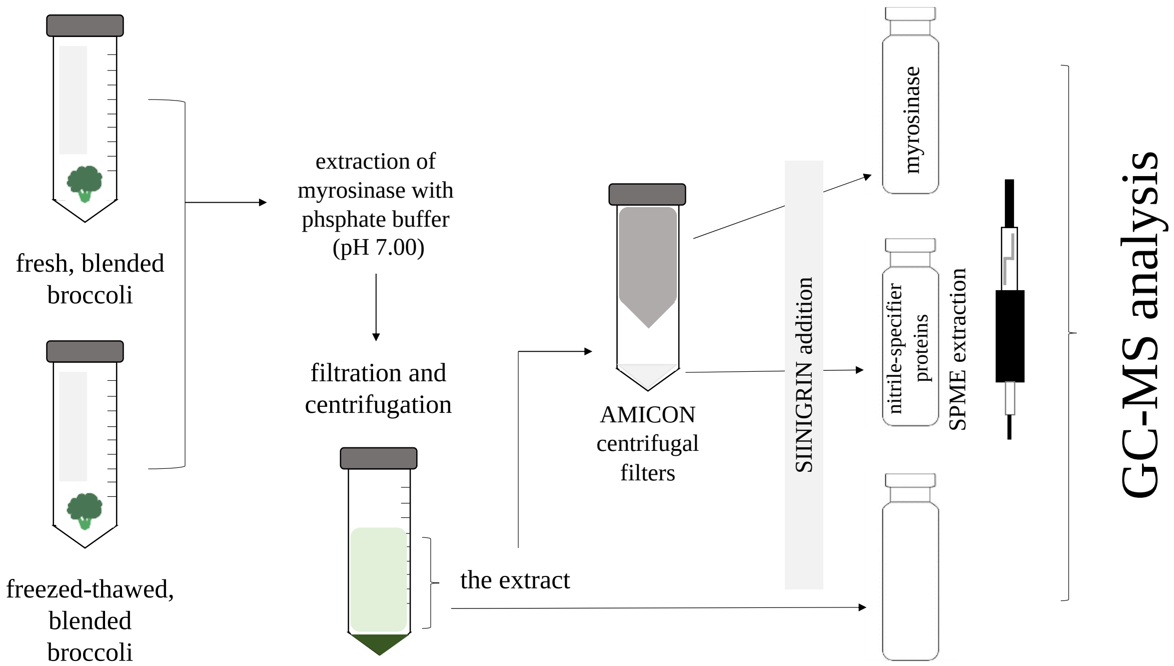

Figure S4. The scheme of enzymes activity assay experiment.

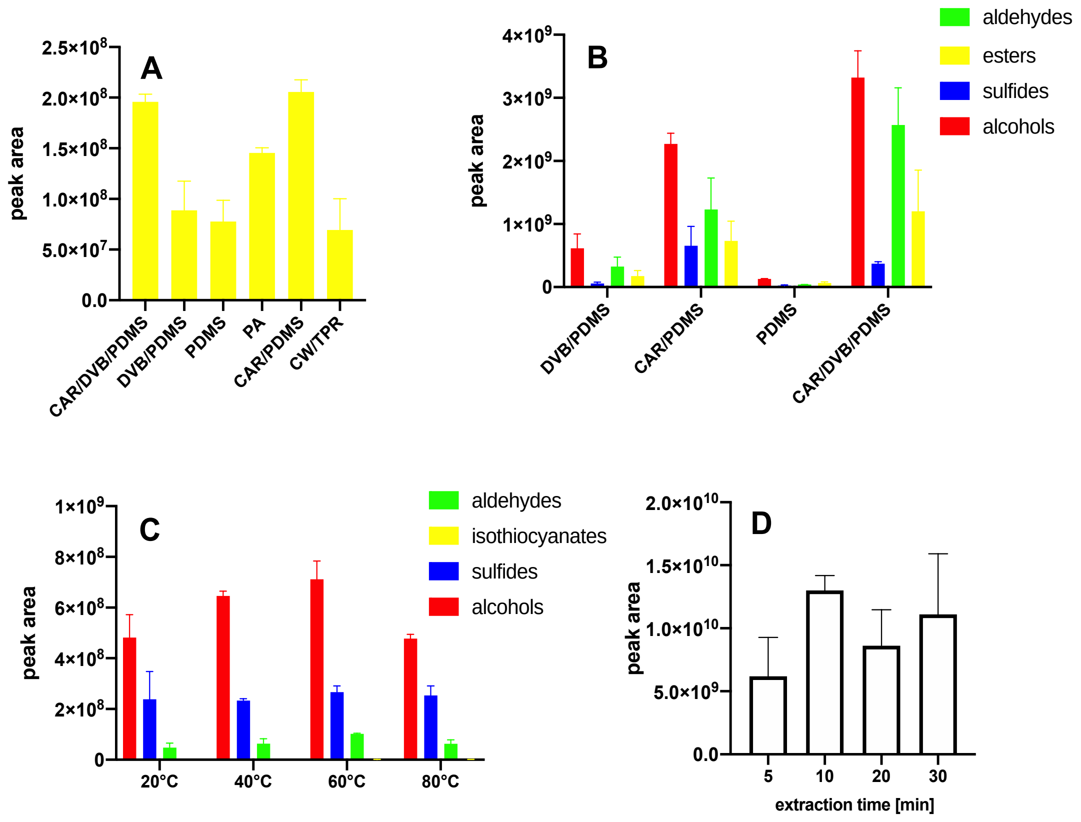

Figure S5. SPME extraction parameters investigated for sampling of broccoli volatiles. A) – fiber effectiveness in extraction of isothiocyanates from standards solution; B) – fiber effectiveness in extraction of volatiles from broccoli; C) – influence of extraction temperature on the amount of volatiles isolated from broccoli; D) – influence of extraction time on the amount of volatiles isolated from broccoli.

Table S2. The mean values and standard deviations of the measured geometric parameters for all sample treatment methods for imaging from the parenchymatic tissue area.

		Cells		All objects	
		Avg.	Std.dev.	Avg.	Std.dev.
Area (µm^2)	Control	1580,00	717,10	1573,64	793,00
	Fast freezing	1619,00	689,61	1742,00	968,71
	Slow freezing	1567,29	865,65	1911,73	2904,18
Perimeter (µm)	Control	162,99	39,44	163,77	45,37
	Fast freezing	168,81	37,57	174,46	54,28
	Slow freezing	165,43	49,95	182,98	108,60
Elongation (a.u.)	Control	1,43	0,30	1,46	0,32
	Fast freezing	1,42	0,28	1,46	0,32
	Slow freezing	1,52	0,38	1,57	0,44
Circularity (a.u.)	Control	0,71	0,07	0,70	0,08
	Fast freezing	0,71	0,07	0,69	0,09
	Slow freezing	0,68	0,09	0,65	0,11

Table S3. The mean values and standard deviations of the measured geometric parameters for all sample treatment methods for imaging from the epidermal tissue area.

		Cells		All objects	
		Avg.	Std.dev.	Avg.	Std.dev.
Area (µm^2)	Control	373,01	239,36	376,47	246,92
	Fast freezing	370,63	243,90	362,04	250,44
	Slow freezing	475,96	296,05	522,53	470,66
Perimeter (µm)	Control	75,28	25,57	76,34	27,10
	Fast freezing	75,26	25,87	75,31	27,69
	Slow freezing	87,36	29,68	95,42	55,30
Elongation (a.u.)	Control	1,40	0,32	1,44	0,38
	Fast freezing	1,42	0,33	1,48	0,41
	Slow freezing	1,47	0,39	1,60	0,73
Circularity (a.u.)	Control	0,76	0,09	0,75	0,10
	Fast freezing	0,75	0,10	0,74	0,12
	Slow freezing	0,73	0,10	0,70	0,14

Table S4. Influence freezing-thawing of broccoli florets on extracted volatiles. Values for $p < 0.05$ are considered significant difference

	peak area ± standard deviation						
	fresh			frozen-thawed			***p*-value**
alcohols	6E+08	±	1E+07	2,9E+08	±	7E+07	**0,00005**
aldehydes	5E+07	±	8E+06	1,9E+08	±	4E+07	**0,178**
tiocyanates	3E+08	±	7E+07	8,9E+07	±	5E+06	**0,021**
nitriles	2E+06	±	8E+05	1,6E+08	±	4E+07	**0,0026**

Table S5. Influence of broccoli florets preparation on the amount of extracted volatiles by SPME. Values for $p < 0.05$ are considered significant difference

	peak area ± standard deviation			
	cutting	blending	shredding	***p*-value**
alcohols	1E+08 ± 2E+07	4E+08 ± 7E+06	3E+08 ± 2E+07	**0,003**
isothiocyanates	9E+06 ± 1E+06	3E+08 ± 7E+07	1E+08 ± 2E+07	**0,02**
sulfides	1E+08 ± 2E+07	2E+08 ± 3E+07	2E+08 ± 2E+07	**0,25**
aldehydes	5E+06 ± 1E+06	5E+07 ± 9E+06	2E+07 2E+06	**0,002**

Table S6 Statistical results for the comparison of sample pairs (from Tab.S3) using Tukey's Multiple Comparison Test. The table reports ***p* value**. Values for $p < 0.05$ are considered significant difference

sample pairs	alcohols	isothiocyanates	sulfides	aldehydes
cutting-blending	0.00016	0.00081	-	0.000095
shredding-blending	0.007	0.005	-	0.0006
shredding-cutting	0.006	0.153	-	0.06

"-" p value >0.05 according to Tab.S3.

Table S7 Influence of enzymatic reactions quenching in broccoli florets on extracted volatiles. Values for $p < 0.05$ are considered significant difference

	LN	EDTA	cooking	no action	***p*-value**
sulfur	3,00E+07 ± 3,00E+06	7,00E+06 ± 2,00E+06	6,00E+07 ± 3,00E+06	3,00E+08 ± 2,00E+07	**0.005**
aldehydes	2,00E+08 ± 1,00E+07	3,00E+08 ± 3,00E+06	3,00E+06 ± 1,00E+05	9,00E+06 ± 2,00E+06	**0.0009**
alcohols	1,00E+08 ± 7,00E+05	1,00E+08 ± 856889	4,00E+05 ± 38383	3,00E+08 ± 891235	**0.0001**
ITC	1,00E+07 ± 2,00E+06	6,00E+06 ± 747805	6321 ± 3449	1,00E+07 ± 5,00E+06	**0.045**
furan, 2-ethyl	1,00E+08 ± 1,00E+06	4,00E+07 ± 3,00E+06	3,00E+05 ± 45410	4,00E+06 ± 172172	**0.0004**

Table S8. Statistical results for the comparison of sample pairs (from Tab.S5) using Tukey's Multiple Comparison Test. The table reports *p* value. Values for $p < 0.05$ are considered significant difference

sample pairs	sulfides	aldehydes	alcohols	isothiocyanates	furan, 2-ethyl
no action - LN	0.00008	0.000097	1E-10	0.58	0
cooking - LN	0.14	0.000086	1E-10	0.047	0
EDTA - LN	0.23	0.0008	0.007	0.32	0
cooking - no action	0.0001	0.8	1E-10	0.028	0.29
EDTA - no action	0.00006	0.00001	1E-10	0.093	0.0001
EDTA - cooking	0.02	0.00001	1E-10	0.27	0.00008
